# Carbon dots derived from folic acid attenuates osteoarthritis by protecting chondrocytes through NF-κB/MAPK pathway and reprogramming macrophages

**DOI:** 10.1186/s12951-022-01681-6

**Published:** 2022-11-03

**Authors:** Yu Jin, Qing Zhang, Xing Qin, Zhen Liu, Zhenxia Li, Xiaoxia Zhong, Lunguo Xia, Jie He, Bing Fang

**Affiliations:** 1 Department of Orthodontics, Shanghai Ninth People’s Hospital, Shanghai Jiao Tong University School of Medicine, College of Stomatology, Shanghai Jiao Tong University, National Center for Stomatology, National Clinical Research Center for Oral Diseases, Shanghai Key Laboratory of Stomatology, 500 Quxi Road, Shanghai, 200011 China; 2 State Key Laboratory of Advanced Optical Communication Systems and Networks, Key Laboratory for Laser Plasmas (Ministry of Education), School of Physics and Astronomy, Shanghai Jiao Tong University, No. 800, Dongchuan Road, Minhang District, Shanghai, 200240 China; 3 Department of Oral and Maxillofacial-Head and Neck Surgery, Shanghai Ninth People’s Hospital, Shanghai Jiao Tong University School of Medicine, College of Stomatology, Shanghai Jiao Tong University, National Center for Stomatology, National Clinical Research Center for Oral Diseases, Shanghai Key Laboratory of Stomatology, 639 Zhizaoju Road, Shanghai, 200011 China

**Keywords:** Osteoarthritis, Carbon dots, Macrophage, Oxidative stress, Cartilage degeneration

## Abstract

**Background:**

Osteoarthritis (OA) is a common joint disorder worldwide which causes great health and economic burden. However, there remains an unmet goal to develop an effective therapeutic method to prevent or delay OA. Chondrocytes, as the major cells involved in OA progression, may serve as a promising therapeutic target.

**Results:**

A kind of carbon dots (CDs) with excellent biocompatibility was fabricated from folic acid via hydrothermal method and could effectively attenuate osteoarthritis. It was demonstrated that CDs treatment could rescue IL1β-induced proinflammatory responses, oxidative stress, cartilage degeneration and extracellular matrix degradation. Moreover, CDs reprogrammed lipopolysaccharide (LPS)-induced macrophage inflammation and polarization. Conditioned medium (CM) from CDs-treated macrophages could attenuate IL1β-induced chondrocyte injury. Also, CM from CDs-treated chondrocytes had immunoregulatory functions on macrophages. Mechanistically, CDs inhibited the activation of nuclear factor-κB (NF-κB) and mitogen-activated protein kinases (MAPK) signaling pathways in IL1β-stimulated chondrocytes. In vivo, anterior cruciate ligament transection (ACLT) mice model was adopted and it was indicated that intra-articular injection of CDs effectively delays OA pathogenesis.

**Conclusions:**

Taken together, these findings indicated CDs could mediate OA via promoting cartilage repair and immunomodulating macrophages within local microenvironment, which may provide evidences for utilizing CDs as a novel nanomaterial for OA treatment.

**Supplementary Information:**

The online version contains supplementary material available at 10.1186/s12951-022-01681-6.

## Introduction

Osteoarthritis (OA) is a complex musculoskeletal disease afflicts patients with physical and mental health and lay great economic burden on society [1]. Pathologically, OA is primarily characterized by cartilage degradation, synovitis inflammation, and subchondral bone damage [2]. However, there is still an unmet goal for treating OA and current strategies could only relieve symptoms [3]. Therefore, a comprehensive understanding of potential mechanisms underlying OA progression and developing therapeutic targets to alleviate OA pathogenesis is greatly needed.

The etiology for OA is complicated while inflammatory environment was reported to be crucial in the initiation and progression of OA [1]. An elevated level of interleukin-1β (IL-1β) was observed in the synovial fluid from OA patients [4]. Also, multiple studies have reported the ability of IL-1β to stimulate inflammatory reactions, mediate cartilage matrix degradation, consequently disturbing cartilage homeostasis [5]. Chondrocytes, as the main cells in the articular cartilage, are mostly subjected to external stimuli and their abnormal functions or impaired survival may initiate OA pathogenesis [6]. Thus, the development of novel regents which could rescue IL-1β-induced chondrocyte dysfunction may be helpful in OA therapy.

Emerging evidences have uncovered the significance of macrophages in various chronic diseases, including rheumatoid arthritis and OA [7, 8]. It was suggested that macrophages play critical roles in the inflammatory and destructive responses during OA [9]. Also, macrophages may serve as crucial mediators in immune defense and tissue repair process [10]. Briefly, there are two major phenotypes, termed M1 macrophage or M2 macrophage, under different conditions. The imbalance of M1/M2 macrophage polarization was proved to participate in various diseases [11]. M1 macrophages are activated by external stimuli such as bacterial lipopolysaccharides (LPS), then produce various proinflammatory cytokines and exacerbate cartilage matrix degradation. It was indicated that activated macrophages contribute to OA initiation and facilitate disease progression [12]. In contrast, M2 macrophages are classified as anti-inflammatory macrophage closely associated with tissue repair [13]. Therefore, we aimed to develop innovative biomaterials that could alleviate inflammatory reactions caused by M1 macrophages and reprogram macrophages from M1 to M2 phenotypic transition, which may be beneficial for OA treatment.

Nowadays, nanomaterials have been widely applied in various fields among which carbon dots (CDs) received more and more attention [14]. CDs have outstanding properties such as good biocompatibility, small size, facile preparation, low cost and excellent optical properties [15–17]. More importantly, CDs derived from raw materials could not only retain the primitive functions of their precursors, but also improve the effect or even possess other properties. For instance, it was reported that CDs derived from onion could act as strong antioxidant factors for diseases [18]. CDs fabricated from adenosine and aspirin had improved osteogenesis-promoting effect [19]. Moreover, CDs prepared from zinc gluconate had higher biocompatibility and greater osteogenic ability [20]. Nevertheless, the therapeutic applications of CDs in OA are rarely investigated. Recently, emerging studies have reported that folate receptors (FRs) are overexpressed on inflammatory or malignant cells while expressed at low levels in normal cells [21]. Some researchers have utilized this feature to develop biomaterials which was combined with folic acid so as to improve the targeting efficiency and therapeutic effect [22, 23]. Given the fact that activated macrophages within inflamed joint areas also express abundant FRs on their surface [24, 25], we determined to fabricate CDs from folic acid to detect its potential therapeutic effect on OA pathogenesis.

Collectively, we synthesized novel folic acid CDs via hydrothermal method and conducted a series of experiments to evaluate its potential for cartilage repair in OA. The results demonstrated that CDs could effectively alleviate OA progression through mediating inflammatory responses, oxidative stress, cartilage degeneration and extracellular matrix degradation. Moreover, CDs had the immuno-modulatory effect to reprogram LPS-induced macrophage inflammation and polarization. In depth mechanistic investigation indicated the modulatory function of CDs may be achieved through targeting NF-κB and MAPK signaling pathways. Finally, we confirmed the therapeutic effect of CDs in ACLT-induced OA model. Taken together, our work provides a new promising biomaterial to treat OA through mediating cartilage homeostasis and immunomodulating macrophages.

## Materials and methods

### Synthesis and of characterization CDs

Specifically, 300 mg folic acid were added into 10 mL deionized water and then dissolved by dropwise adding the NaOH at concentration of 1 M. Rection was performed in polytetrafluoroethylene autoclaves at 140 °C for 6 h. After being centrifuged at 6000 rpm for 15 min, solution was filtrated through 22 μm ultrafiltration membrane. After cooling down at room temperature, the obtained clear brownish black solution was dialyzed through deionized water for 24 h. The MWCO of dialysis bag is 1000 D and the water is changed every 2 h. Finally, CDs we fabricated underwent lyophilization in the freezer dryer for 4 days. For the characterization of CDs, transmission electron microscope (JEOL, JEM-2100F, Japan) was firstly used to manifest the morphology structure of CDs. Particle components analysis was performed by XPS (AXIS ULTRA DLD, Kratos, Japan). Optical vibration spectra was analyzed by UV–vis absorption spectroscopy (Avaspec-2048-2-USB2, Avantes, Netherlands) and Fourier transform infrared spectrometer (Nicolet 6700, Thermo Scientific, USA). Chemical groups were determined by 600 MHz nuclear magnetic (Avance III 600 MHz, Germany) and FTIR (Nicolet 6700, Thermo Scientific, USA).

### Cell culture and treatment

Primary chondrocytes were isolated from C57BL/6 J mice (3-week-old). Firstly, cartilage tissues were obtained and digested in 0.25% trypsin–EDTA for 30 min at 37 °C. Cartilage tissues were then cut into 1 mm^3^ pieces and digested in 0.02% collagenase II for approximately 3 h at 37 °C. Afterwards, chondrocytes were collected and resuspended in DMEM/F12 (Gibco, USA) containing 10% fetal bovine serum (Gibco, AUS) and 1% penicillin–streptomycin (Gibco, USA). Primary chondrocytes from passage 1–3 were used for the following experiments. RAW264.7 macrophage cell line was purchased from the American Type Culture Collection (ATCC, USA) and cultured in DMEM (Gibco, USA) with the same additives. Primary chondrocytes were stimulated with IL-1β (10 ng/mL, Peprotech, USA) to simulate in vitro OA environment. LPS (100 ng/mL, InvivoGen, USA) was added into RAW264.7 murine macrophages for 24 h to polarize cells to M1 type.

### Cellular uptake assay

To investigate cell uptake of CDs, chondrocytes or RAW264.7 murine macrophages with different treatment were cultured with CDs (0.2 mg/mL) for 24 h. Cells were washed by PBS to remove dissociative CDs and then observed with an inverted fluorescence microscope (ZEISS, Germany).

### Cell cytotoxicity assay

A total of 3 × 10^3^ chondrocytes or 8 × 10^3^ macrophages were seeded onto 96-well plates and treated with different concentrations of CDs for consecutive 3 days. At indicated time, cells were incubated with 10% CCK8 solution (New Cell & Molecular Biotech, China) for 2 h. An absorbance of 450 nm was read an ELX Ultra microplate reader (BioTek, USA).

### Live & dead staining

Live & dead staining kit (BestBio, China) was utilized to manifest live or dead cells. Cells were washed by PBS and stained by Calcein-AM and PI solution in serum-free culture medium for approximately 20 min. Stained samples were observed and images were captured under an inverted fluorescence microscope (ZEISS, Germany). Green indicates live cells and red indicates dead cells.

### Cell proliferation assay

Chondrocytes were seeded onto 96-well plates at a density of 3 × 10^3^ cells per well and stimulated with or without IL-1β for 24 h. Subsequently, cells were incubated with different concentrations of CDs and CCK8 assay was performed at 24 h, 48 h, and 72 h after CDs treatment.

### Ethynyl-2′-deoxyuridine (EdU) staining

EdU staining was completed by Cell-Light EdU Apollo567 In Vitro Kit (RIBOBIO, China) as instructions stated. Chondrocytes were reacted with 50 μM EdU solution for 2 h and fixed by 4% paraformaldehyde. Afterwards, chondrocytes were treated with 0.5% TritonX-100 and incubated with Apollo reaction solution for 30 min protected from light. Hoechst 33,342 was utilized to manifest cell nuclei and images were captured by an inverted fluorescence microscope (ZEISS, Germany).

### ROS detection

Intracellular ROS level of different samples was determined by a ROS kit (Beyotime, China). Chondrocytes were labeled with DCFH-DA for 20 min at 37 °C incubator and then washed by PBS for three times. Fluorescence signals were observed and captured by an inverted fluorescence microscope (ZEISS, Germany).

### JC-1 staining

To detect whether chondrocytes have mitochondrial dysfunction, JC-1 staining (Beyotime, China) was performed as instructions indicated. Specifically, chondrocytes were incubated with JC-1 working solution for 20 min at 37 °C and washed with JC-1 buffer for three times. Images were observed and captured under an inverted fluorescence microscope (ZEISS, Germany). A decreased ratio of red fluorescence intensity to green one indicates mitochondrial dysfunction.

### ATP level assay

ATP level was assessed using an ATP assay kit (Beyotime, China). Chondrocytes were washed by PBS and lysis buffer was added into the culture plate. After being centrifuged at 12,000*g* for 5 min at 4 °C, 50 μL supernatant from each group was extracted and mixed with 100 μL ATP detection solution to detect luminescence intensity by a fluorescence microplate reader.

### RNA isolation and RT-qPCR

RNA was extracted by TRIzol reagent (Takara, Japan) and reverse transcription was performed by PrimeScript (Takara, Japan). Quantitative reverse transcriptase-polymerase chain reaction (qRT-PCR) was performed on a Light Cycler 480 II (Roche) with Hieff UNICON^®^ qPCR SYBR Green Master Mix (YEASEN, China). All the primers were synthetized by Sangon Biotech (Shanghai) and the detailed sequences were listed in Additional file 1: Table S1. Relative gene expression was analyzed by 2^−ΔΔCt^ method with β-actin acting as the housekeeping gene.

### Immunofluorescence staining

Cells were immobilized by 4% paraformaldehyde and permeabilized with 0.1% Triton X-100. The fixed cells were incubated with primary antibodies against iNOS (proteintech, USA), COX-2 (CST, UK), COLII (proteintech, USA), MMP13 (proteintech, USA), p65 (CST, UK), CD68 (proteintech, USA), Arg1 (Abclonal, USA) and CD206 (proteintech, USA) at 4 °C overnight. Next day, cells were stained with Alexa Fluor 488-conjugated or Alexa Fluor 647-conjugated secondary antibodies (Abcam, USA) protected from light. Finally, cell nuclei was manifested by DAPI (YEASEN, China). Fluorescence microscope (ZEISS, Germany) was used to observe and capture the fluorescence images.

### Western blot

Total cell protein was extracted with SDS lysis buffer (Beyotime, China). Cytoplasmic and nuclear protein extracts from cells were separated by using Nuclear and Cytoplasmic Extraction Reagents (Beyotime, China). Western blot assay was completed as previously described [26]. Primary antibodies against COX-2 (CST, UK), iNOS (CST, UK), COLII (proteintech, USA), MMP13 (proteintech, USA), Nrf2 (proteintech, USA), HO-1 (proteintech, USA), p-p65(CST, UK), p65 (proteintech, UK), p-IκBα (CST, UK), p-ERK(CST, UK), ERK (CST, UK), p-JNK (CST, UK), JNK (CST, UK), p-p38 (CST, UK), p38 (CST, UK), Histone H3 (CST, UK) and β-actin (proteintech, USA) was incubated with membranes at 4 °C overnight. After HRP-conjugated secondary antibodies incubation, protein bands were observed on an Amersham600 Chemiluminescence System. The relative intensity of protein band was analyzed by Image J.

### Conditioned medium (CM) collection

Cell supernatants were collected and centrifugated at 1000*g* for 5 min and stored at − 80 °C until use. CM from different groups were obtained to culture cells to investigate the crosstalk between chondrocytes and macrophages.

### ELISA assay

The concentration of proinflammatory mediators (TNF-α and IL6) in supernatant from different samples was determined by ELISA kit (Multisciences, China) following the manufacturer’s instructions.

### Griess assay

The release of nitric oxide was detected by Griess assay kit (Beyotime, China) as the instructions indicated. The absorbance at 540 nm was measured on an ELX Ultra microplate reader (BioTek, USA).

### In vivo osteoarthritic model

Animal experiments involved in this study were approved by the Ethics Board of Shanghai Ninth People’s Hospital affiliated to Shanghai Jiao Tong University School of Medicine. In the present study, anterior cruciate ligament transection (ACLT) surgery was performed on the left knee of C57BL/6 J mice to construct OA model as previously described [27, 28]. A total of 12 male C57BL/6 J mice (12-week-old) was included and randomly allocated to three groups, with four mice in each group (Sham, ACLT, and ACLT + CDs). Two weeks after ACLT operation, intra-articular injection of 2 mg/mL CDs was performed on ACLT + CDs group twice per week for consecutive six weeks. All the mice were sacrificed 8 weeks after surgery operation and tissues were immediately collected for following experiments.

### Histological analysis and immunohistochemistry assay

Collected tissues were immediately fixed in 4% paraformaldehyde for 48 h and then decalcified in 10% EDTA for one month. Samples were embedded in paraffin and slices were obtained at approximately 5 μm thick. For histological analysis, samples were stained with hematoxylin–eosin (HE) and Safranin-O-fast green. Osteoarthritis Research Society International (OARSI) scoring system [29] was used to evaluate OA severity. For immunohistochemistry assay, samples were deparaffinized, dehydrated, and then incubated in 0.3% hydrogen peroxide. Proteinase K incubation method was used for antigen retrieval. Specimen were probed with antibodies of COLII (Proteintech, USA) and MMP13 (Proteintech, USA) at 4 °C overnight. DAB solution was used to stain samples following secondary antibody incubation (Gene Tech, China). Cell nuclei was finally manifested by hematoxylin and pictures were captured on a light microscope (Nikon, Japan). The relative staining intensity was calculated by ImageJ software.

### Statistical analysis

Statistical analysis was performed by GraphPad Prism 8.0 software throughout this study. Analysis of two or more groups was conducted by Student’s t-test or one-way ANOVA and P < 0.05 was considered as statistically significant in all analyses.

## Results

### Synthesis and characterization of CDs

**Fig. 1:**
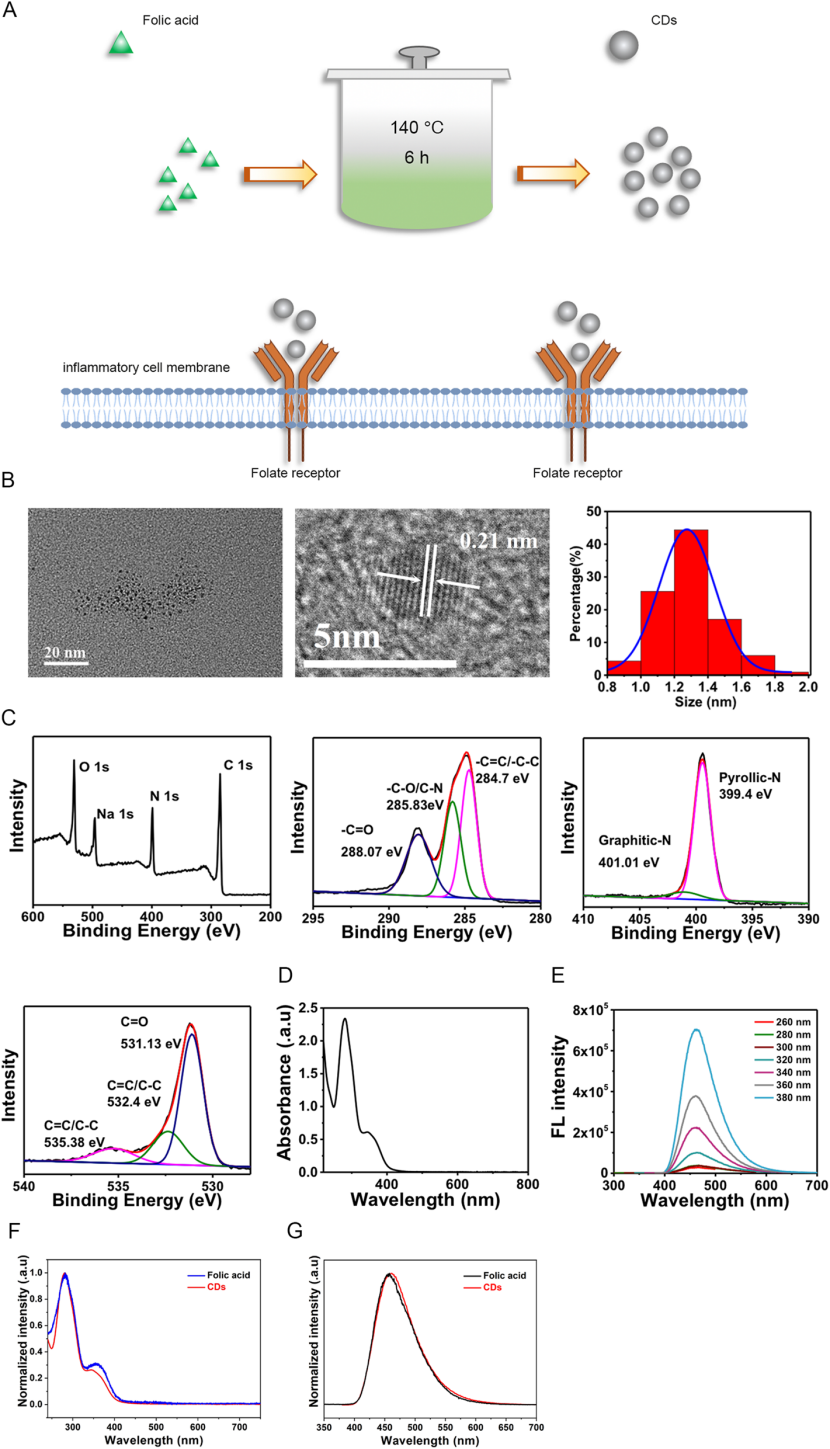
Synthesis and characterization of CDs. **A** Schematic illustration showing the procedures of synthesizing CDs from folic acid. **B** TEM images and size distribution of CDs. **C** Full scan and high-resolution XPS spectra of CDs. **D** UV–vis spectra of CDs. **E** FL spectra of CDs. **F** UV–vis spectra of folic acid and prepared CDs. **G** FL spectra of folic acid and prepared CDs

CDs were synthesized from folic acid through hydrothermal method as described in the Materials and Methods section and the procedures were summarized in Fig. 1A. Transmission electron microscope (TEM) images showed CDs were uniformly dispersed and the particle size distribution (PSD) results indicated CDs were primarily distributed between 1.0 and 1.6 nm (Fig. 1B), which could facilitate its entrance into cells. High-resolution TEM images indicated the lattice distance is 0.21 nm (Fig. 1B). Subsequently, X-ray photoelectron spectroscopy (XPS) assay was conducted to identify chemical composition. Full-scale spectrum illustrated that CDs has a composition of carbon (65.97%), nitrogen (17.95%), and oxygen (16.09%) (Fig. 1C). Moreover, high-resolution spectrum of C 1 s demonstrated the presence of –C=O (288.07 eV), –C–O/C–N (285.83 eV), and –C=C/–C–C (284.7 eV) (Fig. 1C). High-resolution spectrum of N 1 s revealed the presence of two nitrogen species of pyridinic N (399.4 eV) and Graphitic N (401.01 eV) (Fig. 1C). High-resolution O 1 s spectrum indicated the existence of C=O (531.13 eV), C=C/C–C (532.4 eV), and C=C/C–C (535.38 eV) (Fig. 1C). UV–vis absorption spectrum showed that CDs is featured by two absorption bands, one band at 280 nm resulted from π → π* transition and the other one at 320 nm is assigned to n → π* transition (Fig. 1D). FL spectrum results indicated the FL emission of CDs is located at approximately 460 nm, presenting an excitation independent behavior at excitation wavelength ranging from 260 to 380 nm (Fig. 1E). Afterwards, we detected whether prepared CDs maintain the pterin structure of folic acid, which is responsible for the selective binding to the folate receptor. The UV–vis spectra showed that folic acid features two absorption bands in which the absorption band at 280 nm resulted from π → π* transition of para-aminobenzoic acid and the absorption band at 365 nm is assigned to n → π* transition of pterin (Fig. 1F), which is mainly responsible for folate receptor binding [30, 31]. In the case of CDs, absorbance at 280 nm and 365 nm are highly maintained (Fig. 1F). Furthermore, FL comparison were presented in Fig. 1G. When excited at 360 nm, both folic acid and CDs featured similar FL emission at 445 nm. As the FL spectra of folic acid is determined by the structure of pterin, thus, this result also revealed that the pterin responsible for the selective binding to the folate receptor is maintained in the prepared CDs. To further confirm the pterin structure on the CDs, we also performed H NMR and FTIR experiments. As was shown in Additional file 1: Fig. S1, the absorption of folic acid at 3417 cm^−1^ is due to hydroxy (OH) stretching vibration. Absorption peaks at 3542 and 3322 cm^−1^ correspond to stretching vibration of amino groups (–NH). Peaks at 1695 cm^−1^ are attributed to the vibration of carbonyl (–C=O) from carboxylic acid group. Peaks at 1604 cm^−1^ are caused by the bending mode of amino group vibration. Fingerprint of pterin (C3N3) and benzene rings are confirmed by absorption peaks around 1485 cm^−1^. Compared to that of folic acid, CDs features vibration of hydroxy (–OH) at 3415 cm^−1^. The vibration of part –NH groups (3542 and 3322 cm^−1^) and –C=O (1695 cm^−1^) groups disappear. The vibration of structure pterin and benzene rings are highly reserved, which indicates the pterin group of folic acid responsible for folate receptor binding is fully contained within the prepared CDs. H1 NMR spectra of folic acid and CDs were presented Additional file 1: Fig. S2 and S3. It was illustrated that the H atom of pterin ring within folic acid features chemical shifts at 8.65 ppm. Vibration of hydrogen within benzene ring locate at 6.64 ppm and 7.65 ppm. The amino group (–NH_2_) and oxhydryl (–OH) vibration locate at 3.34 ppm and 2.49 ppm (Additional file 1: Fig. S2). In the case of CDs shown in Additional file 1: Fig. S3, the vibration of amino (–NH_2_) and oxhydryl (–OH) groups seems not be affected. While the H atom vibration belongs to pterin and benzene ring largely decreases. This indicates part of the pterin and benzene ring may be destroyed during the preparation. However, by zooming up the picture, it can be observed that the hydrogen vibration belongs to pterin ring and benzene ring locating around 8.65 ppm, 6.64 ppm and 7.65 pm are still reserved. Collectively, the above results indicate that the pterin of folic acid responsible for folate receptor binding retains in the prepared CDs. The findings were consistent with our previous published work and other literatures [32, 33], indicating that the structure of pterin responsible for the folate receptor binding is still residual on the CDs and can also give the CDs excellent targeting ability to the cells with folate receptor overexpression in spite of the high temperature fabrication process.

### Biocompatibility and cellular uptake CDs

**Fig. 2:**
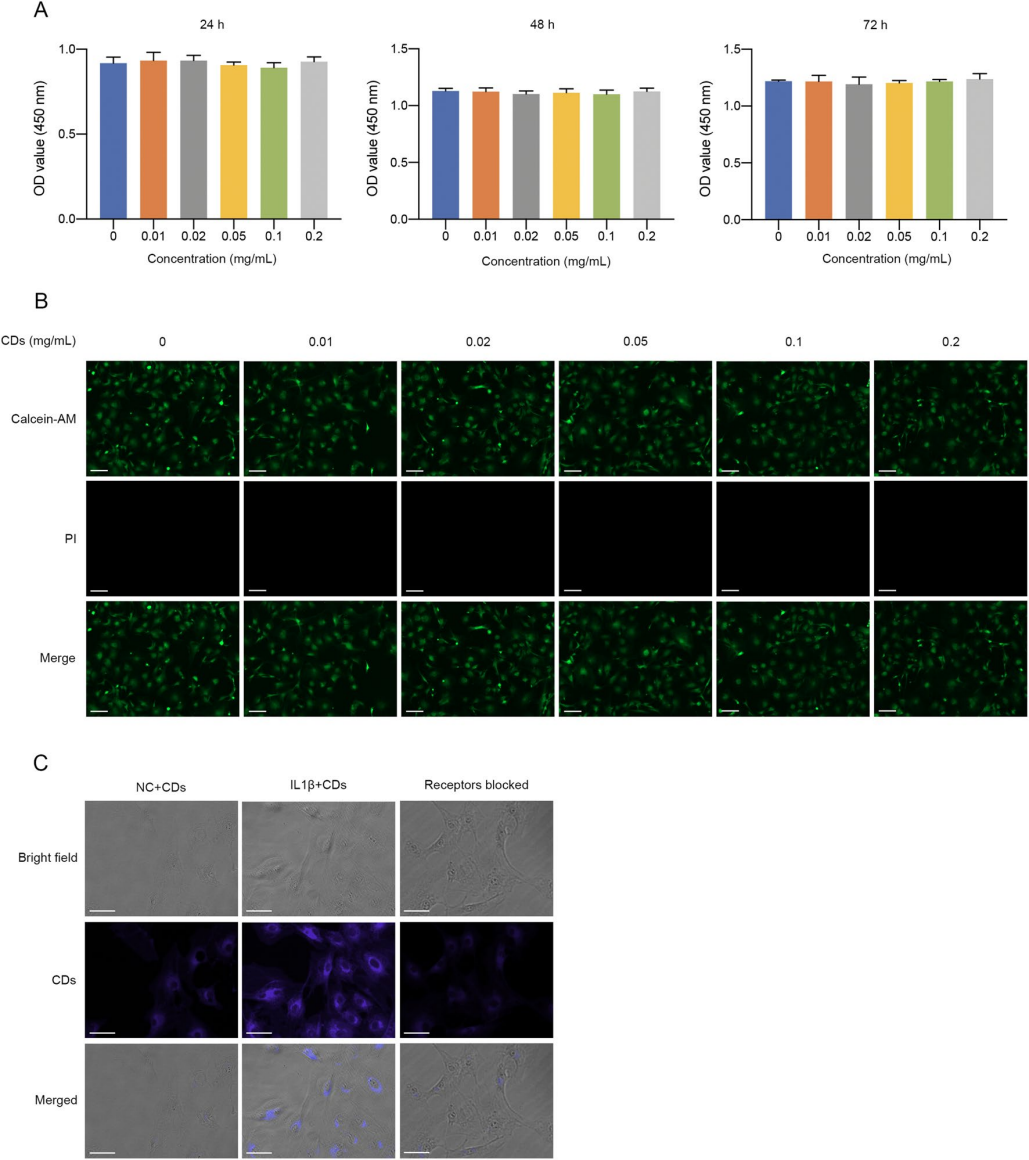
Biocompatibility and cellular uptake CDs in primary chondrocytes. **A** CCK8 assay of chondrocytes with different concentration of CDs incubation. **B** Live & dead staining of chondrocytes incubated with different concentration of CDs for 24 h. Scar bar: 100 μm. **C** Cellular uptake of CDs in chondrocytes with or without IL-1β stimulation, or IL1β-induced chondrocytes with FRs pre-blocked. Scar bar: 50 μm

Biocompatibility of CDs was evaluated by CCK-8 assay. It was shown that different concentration of CDs (up to 0.2 mg/mL) have no evident cytotoxicity on primary chondrocytes or RAW264.7 macrophages (Fig. 2A, Additional file 1: Fig. S4A). Live & dead staining assay also showed no dead cells under CDs treatment (Fig. 2B, Additional file 1: Fig. S4B). Therefore, CDs have excellent biocompatibility and could be used for further biological applications. To observe the cellular uptake of CDs, cells with or without IL-1β/LPS stimulation were cultured with CDs for 24 h. As was shown in Fig. 2C and Additional file 1: Fig. S4C, cells without stimuli have little fluorescence while large amounts of CDs were internalized by cells under inflammatory conditions. From our perspective, the non-opsonic phagocytosis of CDs in macrophages is dependent on the activation status. Next, we wondered whether the cellular uptake of CDs was achieved through FRs on the cell surface, we added excessive folic acid (200 μg/mL) into the culture medium for 2 h after which incubated with CDs. It was indicated that folic acid pretreatment significantly decreased cellular uptake of CDs (Fig. 2C), suggesting that CDs entered into cells via FRs.

### CDs alleviated the effect of IL-1β on chondrocyte proliferation

**Fig. 3:**
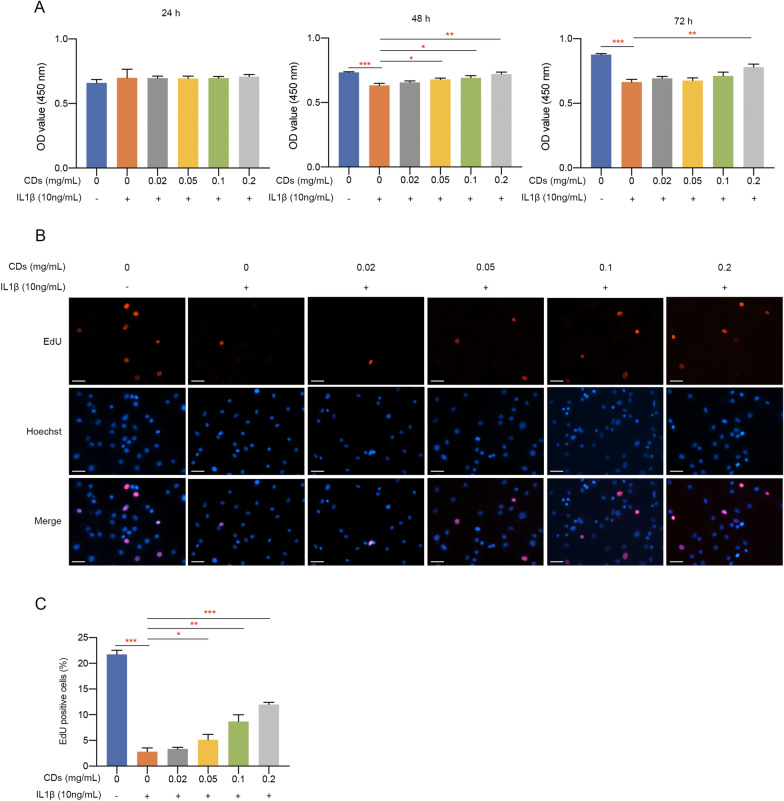
CDs rescued the destructive effect of IL-1β on chondrocyte proliferation and viability. **A** CCK8 assay indicated CDs could alleviate the impact of IL-1β on chondrocyte proliferation. **B** EdU staining showed CDs rescued the decreased cell viability of IL-1β-stimulated chondrocytes. Scar bar: 40 μm. **C** Statistical analysis results of three independent EdU assay experiments by one-way ANOVA method. *p < 0.05; **p < 0.01; ***p < 0.001

We utilized CCK8 assay to investigate the effect of CDs on primary chondrocytes proliferation. It was indicated that IL-1β stimulation significantly suppressed cell proliferation while CDs treatment alleviated this negative impact in dose-dependent manners (Fig. 3A). Subsequently, EdU staining was performed and the results also showed that CDs could partially rescue the decreased cell viability caused by IL-1β (Fig. 3B, C).

### CDs inhibited IL-1β‐induced oxidative stress and mitochondrial dysfunction

**Fig. 4:**
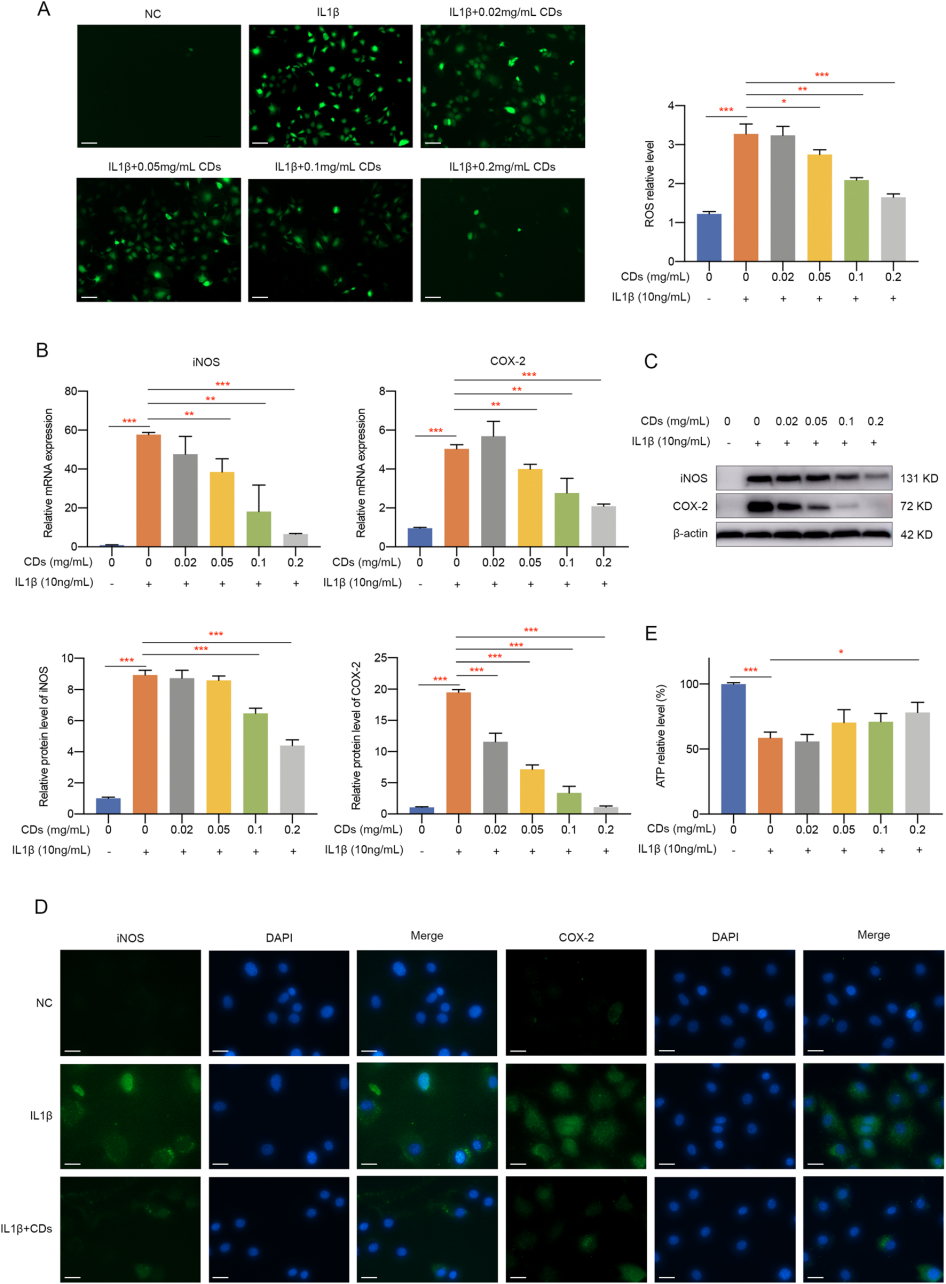
CDs inhibited IL-1β-induced oxidative stress. **A** Intracellular ROS in chondrocytes with different treatment. Scar bar: 100 μm. **B** The elevated level of iNOS and COX-2 in IL-1β-induced chondrocytes was rescued by CDs treatment. **C** Western blot assay indicated CDs could effectively inhibit iNOS and COX-2 expression. **D** Immunostaining assay showed the dysregulation of iNOS and COX-2 was rescued by CDs treatment. Scar bar: 20 μm. **E** The restorative effect of CDs on ATP production in IL-1β-stimulated chondrocytes. *p < 0.05; **p < 0.01; ***p < 0.001

**Fig. 5:**
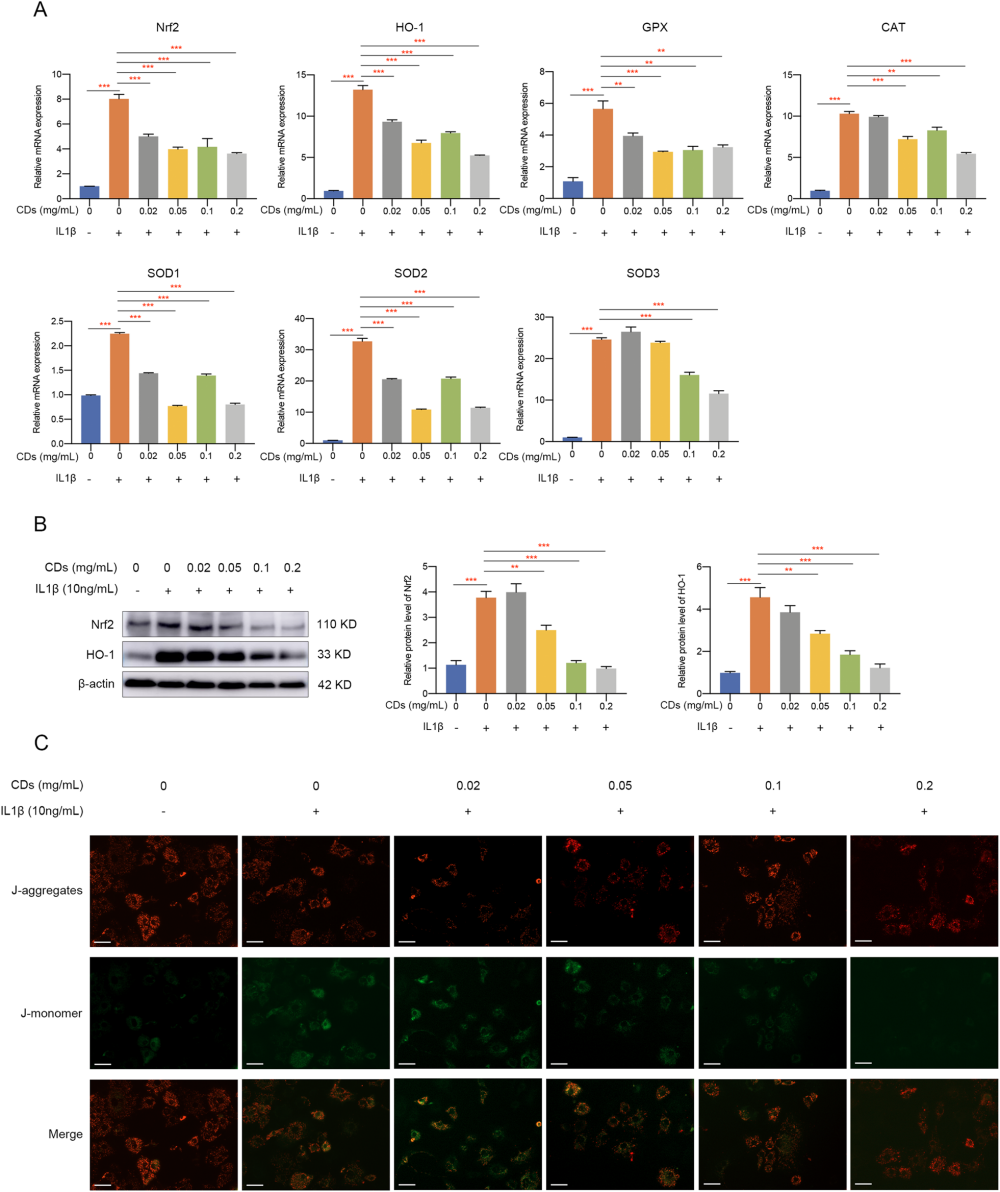
The mediatory functions of CDs on oxidative stress-related genes and mitochondrial dysfunction. **A** Oxidative stress-related genes was verified to be modulated by CDs. **B** CDs exerted its mediatory roles in oxidative stress-induced cartilage injury through targeting Nrf2 signaling pathway. **C** Mitochondrial dysfunction induced by IL-1β was rescued by CDs incubation. Scar bar: 40 μm **p < 0.01; ***p < 0.001

Since oxidative stress exists in OA pathogenesis and its level is closely related with chondrocyte apoptosis, catabolic processes, and matrix degradation, we next investigated whether CDs could mediate IL-1β-stimulated oxidative stress. Immunofluorescence assay showed that the intracellular ROS production was greatly reduced by CDs (Fig. 4A). Also, mRNA level of oxidative-related genes, including iNOS and COX-2, was modulated by CDs (Fig. 4B). Western blot results suggested the dose-dependent inhibitory effect of CDs on iNOS and COX-2 (Fig. 4C). Additionally, immunofluorescence staining showed similar results, with a lower staining intensity of iNOS and COX-2 in chondrocytes with CDs treatment (Fig. 4D). In most cases, ATP production is mediated by oxidative stress. It was manifested in Fig. 4E that the decreased ATP level in activated chondrocytes was dramatically increased after CDs administration. Given the importance of Nrf2/HO-1 signaling in oxidative stress, we determined to examine whether CDs function through mediating Nrf2/HO-1 pathway. Firstly, several oxidative-related markers (Nrf2, HO-1, GPX, CAT, SOD1, SOD2, and SOD3) were evaluated and the results suggested that CDs influence oxidative stress (Fig. 5A). Further experiments showed that IL-1β induced a remarkable increase of Nrf2 and HO-1 in chondrocytes (Fig. 5B). On the contrary, CDs treatment remarkably decreased Nrf2 and HO-1 expression (Fig. 5B). Collectively, our findings implied Nrf2/HO-1signaling pathway may be responsible for CDs-mediated attenuation of the negative impact of IL-1β on chondrocytes. Since mitochondrial dysfunction may occur in inflammation-related diseases, we used fluorescent probe JC-1 to monitor mitochondrial membrane potential under different treatment. Normally, JC-1 presents red fluorescence while once mitochondrial is damaged, the fluorescence will shift from red to green. As was shown in Fig. 5C, mitochondrial membrane potential was impaired after IL-1β stimulation while CDs could rescue this impact.

### CDs protected chondrocytes against IL-1β-induced extracellular matrix degradation and inflammatory responses

**Fig. 6:**
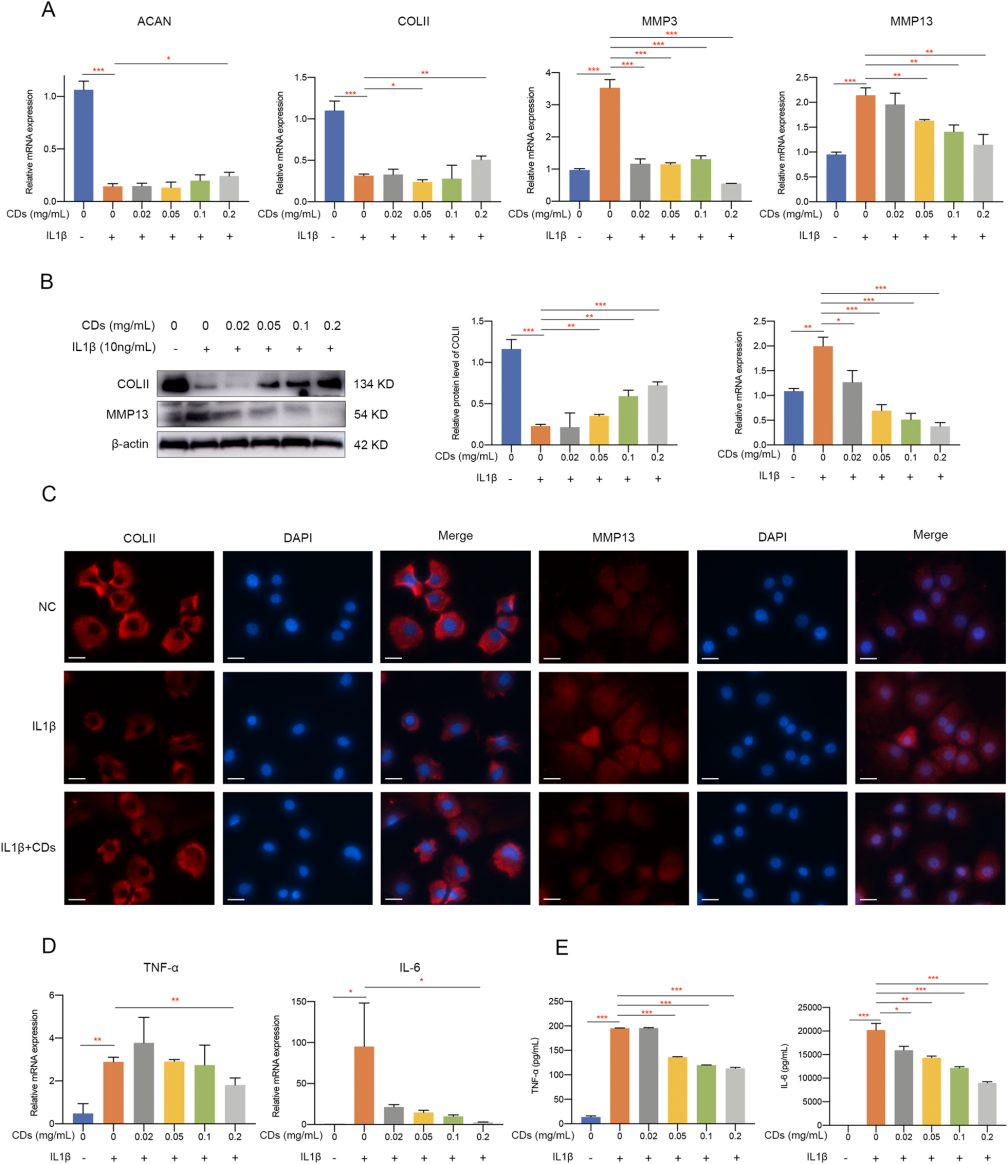
CDs protected chondrocytes against IL-1β-induced extracellular matrix degradation and inflammatory responses. **A** RT-qPCR analysis results showed CDs effectively protected chondrocytes against IL-1β-induced extracellular matrix degradation. **B** The reduced level of COLII and induced level of MMP13 was rescued by CDs treatment. **C** The staining intensity of COLII and MMP13 in IL-1β-stimulated chondrocytes was modulated by CDs. Scar bar: 20 μm. **D** CDs effectively inhibited IL-1β-induced inflammatory responses. **E** The concentration of TNF-α and IL-6 in the supernatant from different groups was determined by ELISA kit. *p < 0.05; **p < 0.01; ***p < 0.001

We implemented a series of experiments to evaluate markers representing ECM degradation in chondrocytes with different treatment. Firstly, qPCR results showed IL-1β treatment remarkably suppressed ACAN and COLII expression while promoted MMP3 and MMP13 expression and these changes were reversed by CDs to a great extent (Fig. 6A). Western blot assay consistently indicated the modulatory effect of CDs on COLII and MMP13 protein expression (Fig. 6B). In addition, immunofluorescence staining demonstrated that CDs significantly alleviated IL-1β-stimulated COLII degradation and MMP13 overexpression in chondrocytes (Fig. 6C). These results suggested the protective role of CDs against IL-1β-induced ECM degradation. Subsequently, the modulatory functions of CDs on IL-1β-induced chondrocyte inflammation was investigated by evaluating proinflammatory mediators. mRNA level of TNF-α and IL-6 was remarkably upregulated upon IL-1β stimulation while CDs treatment attenuated this effect (Fig. 6D). ELISA assay showed consistent results that the induced production of TNF-α and IL-6 was significantly inhibited by CDs (Fig. 6E).

### CDs inhibited NF-κB and MAPK pathway activation in OA chondrocytes

**Fig. 7:**
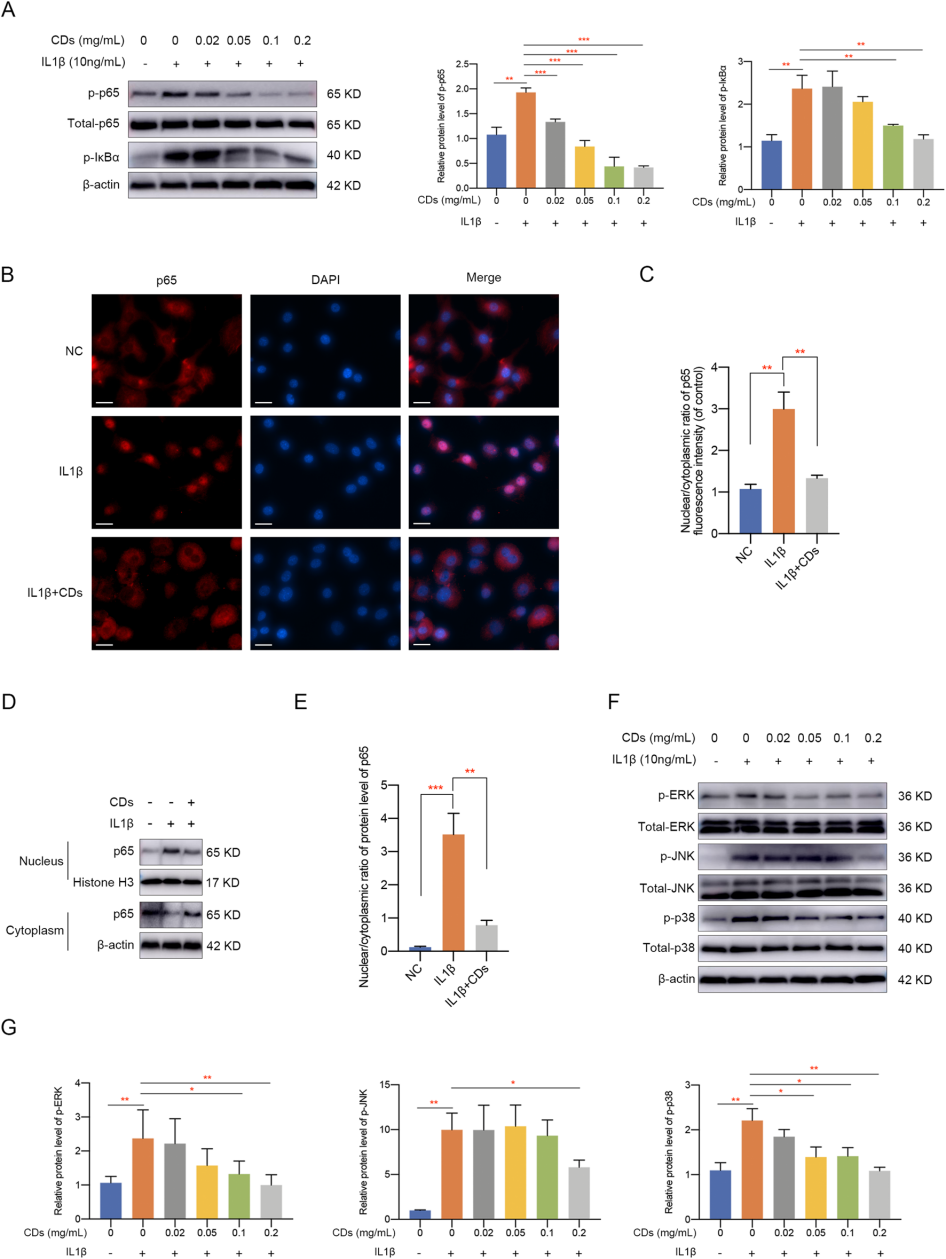
CDs inhibited NF-κB and MAPK pathway activation in OA chondrocytes. **A** Western blot assay indicated CDs inhibited NF-κB pathway activation in OA chondrocytes. **B**, **C** Immunofluorescence assay showed CDs inhibited the nuclear translocation of p65 in chondrocytes with IL-1β stimulation. Scar bar: 20 μm. **D** Western blot assay indicated CDs administration could partially suppress the translocation of p65 from cytoplasm to nucleus caused by IL-1β stimulation. **E** The relative band intensity of p65 in the nucleus relative to the cytoplasm based on Image J software. **F** Western blot assay results indicated CDs inhibited MAPK pathway activation in OA chondrocytes. **G** The relative band intensity of proteins involved in MAPK pathway was calculated by Image J software. *p < 0.05; **p < 0.01; ***p < 0.001

NF-κB and MAPK pathway is widely acknowledged as essential signaling pathways involved in inflammation and multiple diseases [34, 35]. We next intended to explore the potential mechanism underlying CDs-mediated OA pathogenesis. Target proteins in the NF-κB pathway were detected and it was showed that the phosphorylation of p65 and IκBα increased remarkably after IL-1β stimulation while CDs treatment suppressed this effect (Fig. 7A). Immunofluorescence staining results indicated p65 translocated from cytoplasm to nucleus upon IL-1β stimulation while CDs inhibited this trend (Figs. 7B, C). Meanwhile, cytoplasmic and nuclear protein extracts were obtained and western blot assay indicated similar results that CDs administration could partially suppress the translocation of p65 from cytoplasm to nucleus caused by IL-1β stimulation (Fig. 7D, E). Additionally, p-ERK, p-JNK, and p-p38 was consistently activated in IL-1β stimulated chondrocytes and CDs treatment effectively suppressed this activation (Fig. 7F, G). The above results suggested that CDs may regulate OA pathogenesis through NF-κB and MAPK signaling pathways.

### CDs orchestrated macrophage polarization

**Fig. 8:**
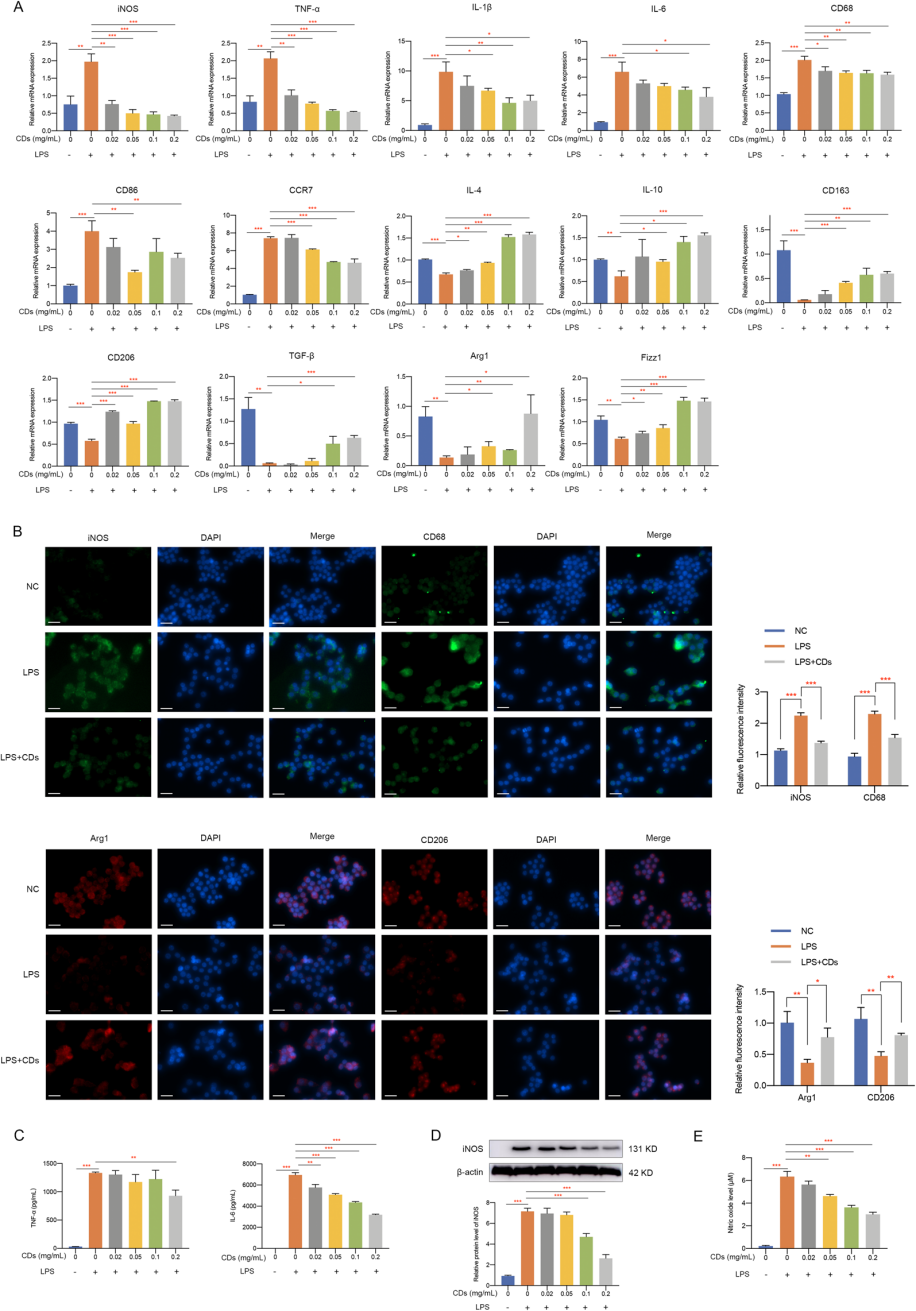
CDs orchestrated macrophage repolarization in vitro. **A** The mRNA level of M1 and M2-related markers in RAW264.7 cells with different treatment. **B** Immunofluorescence staining results indicated CDs incubation could prevent the polarization of macrophages to proinflammatory type. Scar bar: 20 μm. **C** The induced concentration of proinflammatory factors in LPS-induced RAW264.7 cells was suppressed by CDs treatment. **D** The protein level of iNOS in RAW264.7 cells under different treatment. **E** Griess assay was used to evaluate the NO scavenging effects of CDs. *p < 0.05; **p < 0.01; ***p < 0.001

Since more and more researches have uncovered the importance of macrophages in OA [13, 36, 37], we wondered whether CDs we synthesized regulated OA progression through immunoregulatory effect. It was shown in Fig. 8A that an intensive inflammatory response was observed in LPS-stimulated Raw264.7 cells. Moreover, M1-related markers including iNOS, CD68, CD86 and CCR7 were greatly induced in response to LPS stimulation (Fig. 8A). Meanwhile, M2-related markers (IL-4, IL-10, CD163, CD206, TGF-β, Arg1, and Fizz1) were downregulated by LPS (Fig. 8A). After adding different concentrations of CDs into LPS-treated macrophages, it can be observed that CDs was capable of inhibiting various inflammatory factors and reprogramming M1-macrophages in a dose dependent manner (Fig. 8A). Moreover, immunofluorescence staining presented an increased percentage of iNOS and CD68-positive cells in the LPS-treated group while CDs significantly reduced M1 polarization (Fig. 8B). The decreased level of M2-type macrophage markers (Arg1 and CD206) was obviously rescued with the treatment of CDs (Fig. 8B). ELISA assay indicated the elevated concentration of TNF-α and IL-6 was remarkably suppressed by CDs (Fig. 8C). To further evaluate the anti-inflammatory effects of CDs, the protein expression level of iNOS and the release of nitric oxide were detected by western blot assay and Griess assay. The results indicated a significant upregulated protein level of iNOS and a large amount release of nitric oxide upon LPS stimulation, which validated the inflammatory status. Furthermore, the anti-inflammatory effects of CDs could be observed by the downregulated level of iNOS and nitric oxide in a dose-dependent manner (Fig. 8D, E). Collectively, these data demonstrated that CDs could effectively prevent the polarization of RAW264.7 cells to proinflammatory state, so as to attenuate OA progression.

### The crosstalk between macrophages and chondrocytes mediated by CDs

**Fig. 9:**
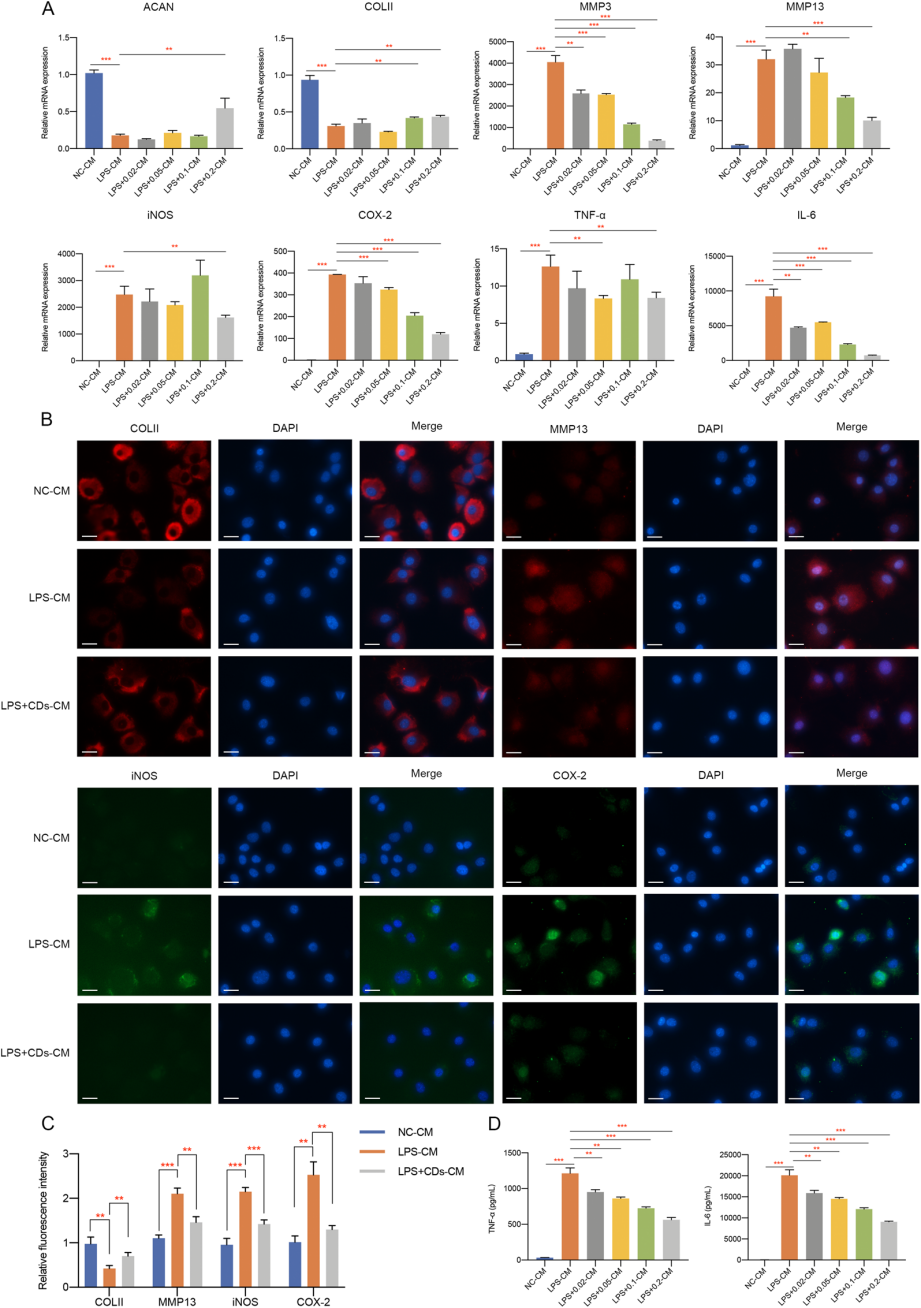
Macrophage CM and co-culture with chondrocytes. **A** The mRNA expression of markers involved in extracellular matrix degradation, oxidative stress, and inflammation in chondrocytes with macrophage CM incubation was determined. **B** Representative markers for cartilage degeneration in macrophage CM-treated chondrocytes was estimated by immunofluorescence assay. Scar bar: 20 μm. **C** The statistical analysis results of fluorescence intensity. **D** The concentration of TNF-α and IL-6 in macrophage CM-treated chondrocytes. **p < 0.01; ***p < 0.001

**Fig. 10:**
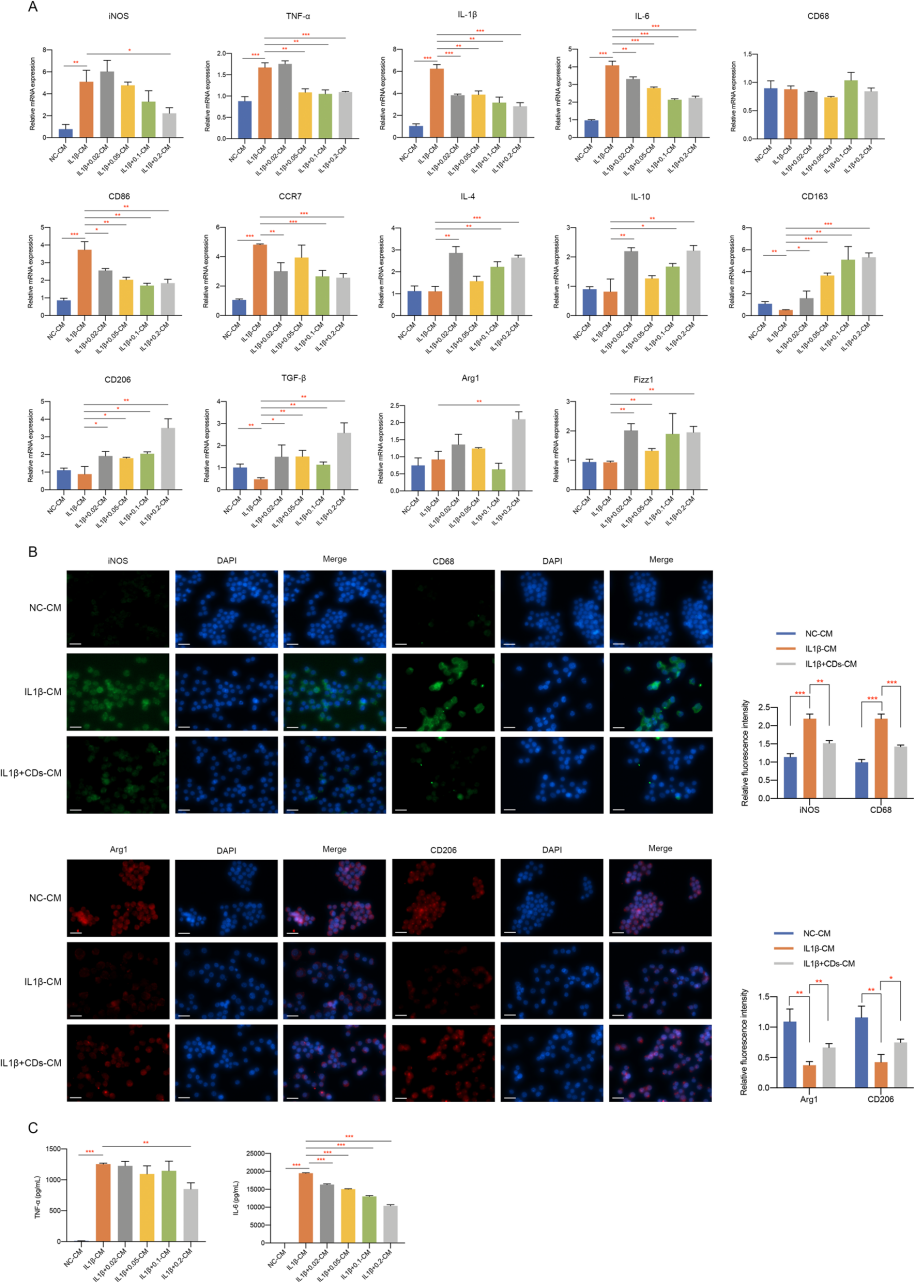
The crosstalk between macrophages and chondrocytes mediated by CDs. **A** The mRNA expression of M1 or M2-realted markers was investigated in chondrocyte CM-treated RAW264.7 cells. **B** The protein expression of iNOS, CD68, Arg1, and CD206 in chondrocyte CM-treated RAW264.7 cells was investigated by immunofluorescence assay. Scar bar: 20 μm. **C** The concentration of proinflammatory mediators in chondrocyte CM-treated RAW264.7 cells. *p < 0.05; **p < 0.01; ***p < 0.001

To examine the relationship between macrophages and chondrocytes mediated by CDs, conditioned medium (CM) was collected and used for the investigation of potential crosstalk between two cell types. Representative markers of inflammation and ECM degradation showed that LPS-CM (CM from LPS-treated macrophages) treated chondrocytes presented a lower expression of ACAN and COLII while highly expressed MMP3, MMP13, iNOS, COX-2, TNF-α and IL-6 (Fig. 9A). CDs treatment significantly alleviated the negative effect of LPS-CM on chondrocytes (Fig. 9A). The same results could be observed by immunofluorescence staining of COLII, MMP13, iNOS, and COX-2 (Fig. 9B, C). ELISA assay also showed CDs administration effectively alleviated the stimulation of TNF-α and IL-6 in chondrocytes under LPS-CM incubation, signifying that CDs may mediate OA through reprogramming macrophages (Fig. 9D). Subsequently, we attempted to detect the potential effect of chondrocytes on macrophages. CM from chondrocytes with different treatment was administrated to RAW264.7 cells and major markers were determined. As was shown in Fig. 10A, markers for M1-like macrophages were upregulated and M2-type-related markers were downregulated in IL-1β-CM treated cells while CM from CDs-treated chondrocytes effectively reprogrammed macrophages. Immunofluorescence assay also showed the regulatory functions of CDs on iNOS, CD68, Arg1 and Fizz1 protein expression in IL-1β-CM treated macrophages (Fig. 10B). The concentration of TNF-α and IL-6 was lower in IL-1β + CDs-CM when compared to that in IL-1β-CM-treated macrophages (Fig. 10C). From above findings, it came to the conclusion that a comprehensive crosstalk between chondrocytes and macrophages occur in CDs-mediated OA pathogenesis.

### CDs alleviated OA progression in vivo

**Fig. 11:**
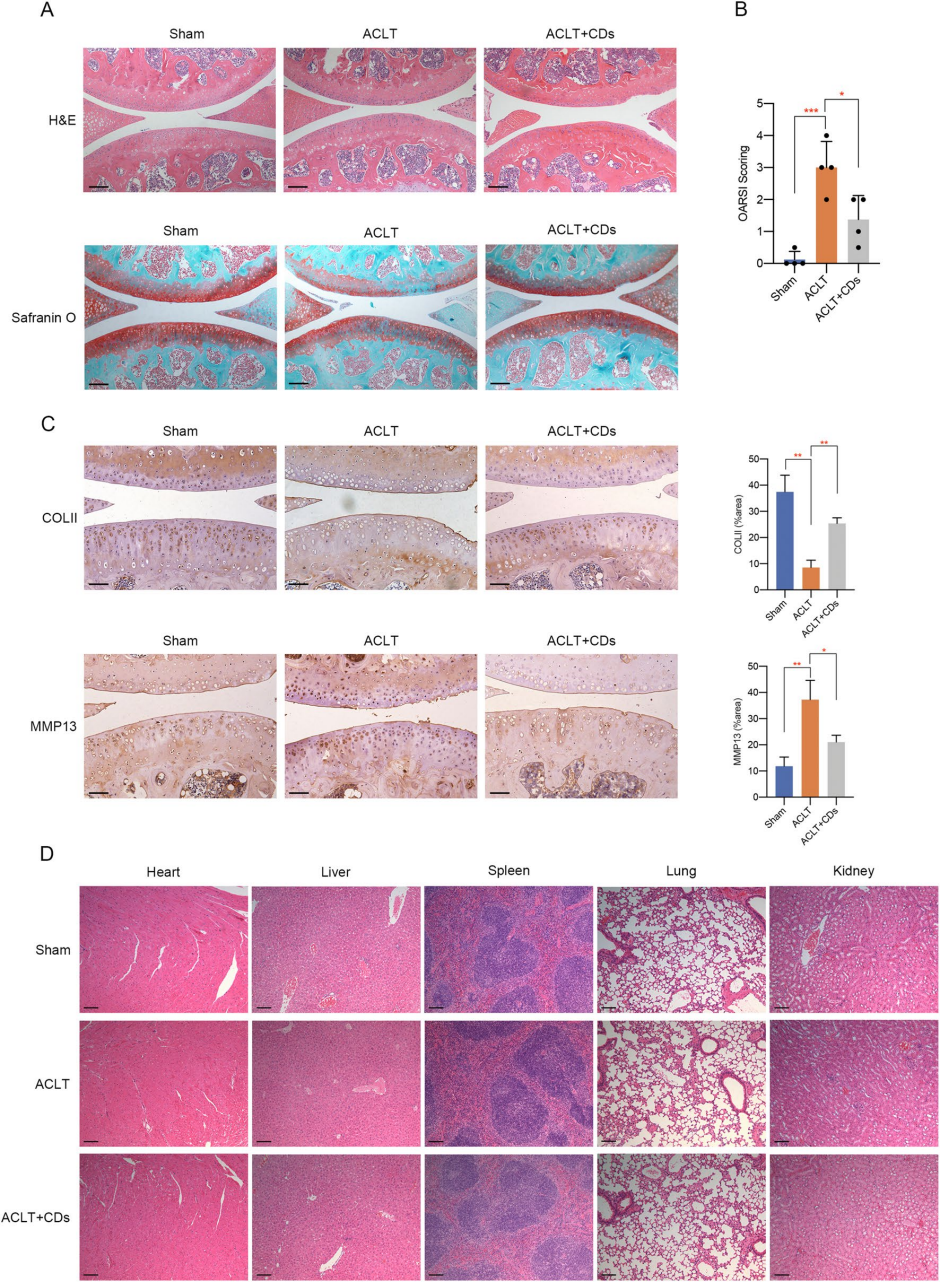
CDs delayed OA progression in vivo. **A** H&E staining and Safranin O and fast green staining results of different samples. **B** OARSI score evaluation in different samples. **C** The staining of COLII and MMP13 and its relative intensity in different samples. **D** H&E staining of heart, liver, spleen, lung, and kidney tissues to examine the toxicity of CDs. Scar bar: 100 μm *p < 0.05; **p < 0.01; ***p < 0.001

**Fig. 12:**
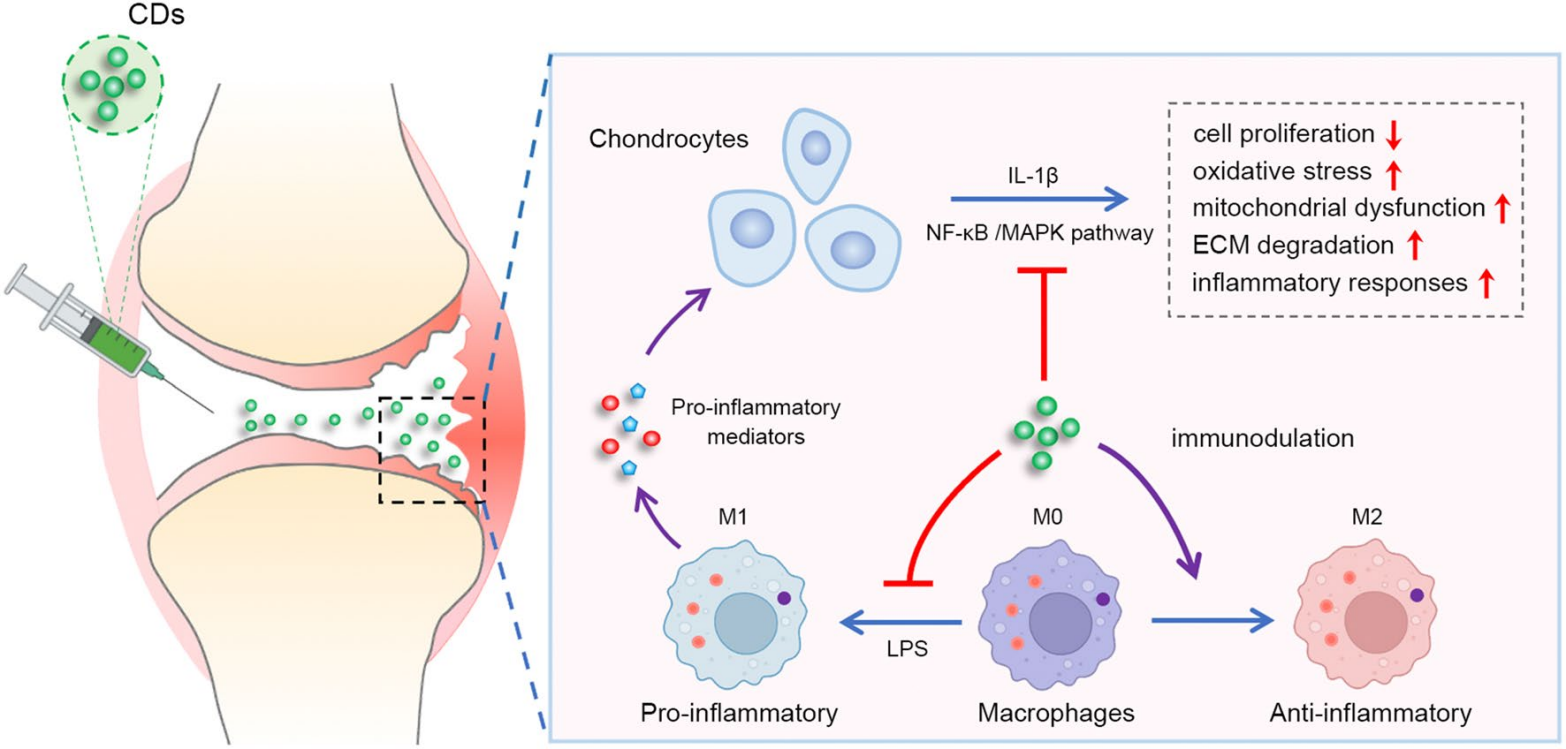
Schematic illustration indicating the immunomodulatory mechanism of CDs-mediated OA therapy

The therapeutic effect of CDs on OA was verified by ACLT mice model. Mice in the ACLT + CDs group were intra-articular injected with 2 mg/mL CDs for consecutive 6 weeks and collected for further analysis. H&E staining and Safranin-O fast green staining showed when compared to Sham group, ACLT group presented higher OARSI score and prominent cartilage defects, while the ACLT + CDs group alleviated the destructive effect (Fig. 11A, B). Moreover, immunohistochemical staining demonstrated a loss of COLII and an overexpression of MMP13 in ACLT group, indicating proteoglycan loss and cartilage matrix degradation (Fig. 11C). CDs administration partially rescued the trend, with a relative higher staining of COLII and lower staining of MMP13 (Fig. 11C). To ensure the safety of CDs for future clinical applications, we finally evaluated in vivo toxicity of CDs on main organs. H&E assay of main organs, that is heart, liver, spleen, lung, and kidney presented no evident pathological changes, indicating that CDs may not produce toxicity or side effects in vivo (Fig. 11D). The overall schematic illustration indicating the immunomodulatory mechanism of CDs-mediated OA therapy was summarized in Fig. 12.

## Discussion

OA is a joint disease whose incidence is increasing and afflicts not only older people but also the young [38]. However, limited to an incomplete understanding of pathological mechanism, OA therapy is still unsatisfactory and only focuses on symptom relief. Existing evidences showed that inflammatory environment is crucial for OA occurrence and development [39]. It was reported that IL-1β is a major pro-inflammatory cytokine in OA and could induce severe inflammatory reactions and cartilage matrix degradation [40, 41]. In the present study, we adopted IL-1β treatment for in vitro assay and demonstrated that CDs could antagonize IL-1β induced production of inflammatory mediators. Besides, IL-1β induced cartilage matrix degradation was remarkably alleviated by CDs.

Recently, a variety of researches have uncovered the significance of excessive oxidative stress in OA, which may result in cartilage degradation and chondrocyte death [42]. Mitochondrial dysfunction caused by aberrant oxidative stress was suggested to be closely associated with cartilage damage. Additionally, chondrocytes within OA environment generate excessive ROS, which may accelerate the pathogenesis process [43, 44]. Specifically, aberrant ROS production could cause chondrocyte death and structural–functional cartilage damage [45, 46]. Therefore, targeting excessive ROS may act as an effective strategy for treating OA. In this work, we validated the chondroprotective effect of CDs and this function may be achieved through decreasing oxidative stress.

As was widely reported, Nrf2 signaling plays essential roles in oxidative stress through regulating detoxifying enzymes and antioxidant proteins [47]. We then intended to clarify the potential mechanism of CDs on alleviating cartilage injury. In agreement with previous studies [48, 49], excessive oxidative stress induced by IL-1β in this study significantly activated Nrf2 signaling in primary chondrocytes. Most published studies suggested Nrf2 signaling activation relates with its protective ability to inflammatory responses [50–52]. Nevertheless, we found that CDs inhibited the activation of Nrf2 signaling and HO-1 expression. From our perspective, although Nrf2 activation is known to protect cells from oxidative stress-related damage, abnormal prolonged Nrf2 activation may conversely cause cell death. Our findings demonstrated that the protective roles of CDs towards oxidative stress-induced OA may result from the downregulation of Nrf2 signaling.

CDs is an emerging nanomaterial with low toxicity and good stability and widely applicated in a variety of frontiers, including cancer therapy, inflammatory diseases, and tissue engineering. It was reported that CDs nanoparticle-embedded hydrogel was able to generate ROS under light irradiation and promote the chondrogenic differentiation of BMSCs [53], implying the prospect of using CDs for cartilage repair. Another study demonstrated that CDs treatment could recover neuron cell viability via eliminating excessive ROS induced by LPS and H_2_O_2_ stimulation, implying that the antioxidative property of CDs renders it as a promising nanodrug for CNS diseases [54]. Moreover, a study fabricated CDs based on citric acid and found its outstanding ability to promote MSCs osteogenic differentiation, which may function through ROS-mediated MAPK pathway [55]. A published study indicated in vivo administration of CDs could attenuate LPS-induced calvarial bone destruction [56]. What is more, CDs derived from metformin remarkably promoted periodontal bone regeneration via modulating ERK/AMPK pathway [17]. CDs fabricated from citric acid and glutathione also presented excellent ROS scavenging ability so as to rescue LPS-induced inflammation in macrophages [57]. In this work, we synthesized novel folic acid-based CDs via hydrothermal method and uncovered its protective roles in OA pathogenesis. Previous studies reported the functions of folic acid to facilitate nanoparticles to accumulate and persist in inflamed tissues, which may result from the overexpressed FRs on activated cells [58]. Some studies even conjugate folate with anti-inflammatory drugs, hydrogels, nanoparticles, or biodegradable polymers to increase the targeting efficiency substantially [59, 60]. The folic acid derived CDs we synthesized in this work could be effectively absorbed by OA chondrocytes or activated macrophages, both were crucial mediators in OA. For OA treatment, a major demand is to increase the penetration of nanomaterials into relative thick cartilage matrix to improve the localization of therapeutics on joint tissues. Studies have demonstrated that substances with relative small size (< 15 nm) could better penetrate the full-depth of articular cartilage and remain much longer time. The CDs we synthesized had a diameter of 0.8–2.0 nm and readily entered into cells in vitro and joint tissues in vivo. A series of experiments confirmed its protective roles towards OA initiation and progression.

Subsequently, we attempted to illustrate the intracellular signaling pathways underlying the therapeutic effect of CDs on OA. It is widely known that NF-κB is closely involved in inflammation-related diseases [61]. Due to its regulation of inflammatory mediators, it was frequently determined as therapeutic target for inflammatory diseases. In normal conditions, the NF-κB dimer binds to IκBα in a state of inactivation while under abnormal conditions, such as exposed to external stimulus or other pro-inflammatory factors, IκBα is degraded by phosphorylation and p65 is translocated from cytoplasm to the nucleus [62]. As a result, we verified the associations between CDs and NF-κB signaling, implying the possible regulatory mechanism of CDs in OA. Furthermore, MAPK pathway, which includes ERK, JNK and p38 pathways, also participates in a variety of physiological and pathological processes. MAPK pathway is activated in OA environment and upregulates the expression of matrix catabolic enzymes and proinflammatory mediators [63]. The present study demonstrated that CDs could also inhibit MAPK pathway activation in OA and this may account for the therapeutic effect of CDs.

Accumulating evidences have illuminated the significance of macrophages in OA inflammation [64]. Macrophages are heterogeneous cells that may present dynamic changes in pathological status. Our study found that CDs could reprogram macrophages by inhibiting pro-inflammatory responses and promoting pro-repair M2 transformation. Then, we attempted to detect the potential interactions between chondrocytes and macrophages within OA microenvironment and the results indicated that LPS-CM from macrophages stimulated severe inflammatory responses and causes cartilage matrix degradation in primary chondrocytes, while LPS + CDs-CM effectively inhibited the destructive effect. Therefore, we suspected that CDs may attenuate OA progression through modulating macrophage polarization. Our findings were consistent with former researches that activated macrophages was reported to aggravate inflammation and promote ECM degradation in chondrocyte microenvironment [65]. Moreover, it was indicated that ECM degradation may in turn act as a stimulus to polarize macrophage polarization [66]. Similarly, our work showed CM from chondrocytes with different treatment regulate macrophage phenotype, suggesting the comprehensive cross-talk between macrophages and chondrocytes. Finally, we validated the in vivo therapeutic effect of CDs on OA pathogenesis and ensured its biological safety, which may provide evidences for further clinical application of CDs in treating OA diseases.

## Conclusion

In summary, we synthesized folic acid CDs and illustrated its functions on attenuating OA progression. We conducted a relative comprehensive analysis of CDs on chondrocytes and macrophages and elucidated their potential close associations during OA pathogenesis. Mechanistic investigation demonstrated that CDs may mediate OA pathogenesis through targeting NF-κB and MAPK pathways. Overall, our work found an innovative immune-modulatory nanodrug which has the potential to be used for OA treatment in the future.

## Supplementary Information

**Additional file 1: Table S1.** The primers used for real-time PCR analysis. **Figure S1.** FTIR spectra of folic acid (FA) and folic acid derived carbon dots (FA-CDs) prepared in this research. **Figure S2.** H^1^ NMR spectra of FA. **Figure S3.** H^1^ NMR spectra of FA-CDs. **Figure S4.** Biocompatibility and cellular uptake CDs in RAW264.7 cells. (A) CCK8 assay of RAW264.7 cells after incubation with different concentration of CDs for 24 h, 48 h, and 72 h. (B) Live & dead staining of RAW264.7 cells incubated with different concentration of CDs for 24 h. Scar bar: 40 μm (C) Cellular uptake of CDs in RAW264.7 cells with or without LPS stimulation, or LPS-induced RAW264.7 cells with FRs pre-blocked. Scar bar: 50 μm.

## Data Availability

All data generated or analyzed during this study are included in this published article and its additional information files.
