# Supplementary material for: Carbon dots derived from folic acid attenuates osteoarthritis by protecting chondrocytes through NF-κB/MAPK pathway and reprogramming macrophages
Source: J Nanobiotechnology. 2022 Nov 3;20:469. doi: 10.1186/s12951-022-01681-6 (PMC9632154; doi:10.1186/s12951-022-01681-6)
Supplement: Supplementary file 1 — Additional file 1: Table S1. The primers used for real-time PCR analysis. Figure S1. FTIR spectra of folic acid (FA) and folic acid derived carbon dots (FA-CDs) prepared in this research. Figure S2. H1 NMR spectra of FA. Figure S3. H1 NMR spectra of FA-CDs. Figure S4. Biocompatibility and cellular uptake CDs in RAW264.7 cells. (A) CCK8 assay of RAW264.7 cells after incubation with different concentration of CDs for 24 h, 48 h, and 72 h. (B) Live & dead staining of RAW264.7 cells incubated with different concentration of CDs for 24 h. Scar bar: 40 μm (C) Cellular uptake of CDs in RAW264.7 cells with or without LPS stimulation, or LPS-induced RAW264.7 cells with FRs pre-blocked. Scar bar: 50 μm. [file 12951_2022_1681_MOESM1_ESM.docx]

**Additional file 1**

**Table S1. The primers used for real-time PCR analysis.**

| **Genes** | **Primer sequences** |
| --- | --- |
| Mouse β-actin | forward 5’-GGCTGTATTCCCCTCCATCG-3’ |
|  | reverse 5’-CCAGTTGGTAACAATGCCATGT-3’ |
| Mouse ACAN | forward 5’-CCTGCTACTTCATCGACCCC-3’ |
|  | reverse 5’-AGATGCTGTTGACTCGAACCT-3’ |
| Mouse COLII | forward 5’-GTGGAGCAGCAAGAGCAAG-3’ |
|  | reverse 5’-CGGAGGAAAGTCATCTGGAC-3’ |
| Mouse iNOS | forward 5’- GTTCTCAGCCCAACAATACAAGA-3’ |
|  | reverse 5’- GTGGACGGGTCGATGTCAC-3’ |
| Mouse COX-2 | forward 5’-GCAGGAAGTCTTTGGTCTGG-3’ |
|  | reverse 5’-AGTTGCTCATCACCCCACTC-3’ |
| Mouse Nrf2 | forward 5’-TCTTGGAGTAAGTCGAGAAGTGT-3’ |
|  | reverse 5’-GTTGAAACTGAGCGAAAAAGGC-3’ |
| Mouse HO-1 | forward 5’-AAGCCGAGAATGCTGAGTTCA-3’ |
|  | reverse 5’-GCCGTGTAGATATGGTACAAGGA-3’ |
| Mouse GPX | forward 5’-CCTTTTAAGCAGTATGCAGGCA-3’ |
|  | reverse 5’-CAAGCCAAATGGCCCAAGTT-3’ |
| Mouse CAT | forward 5’-AGCGACCAGATGAAGCAGTG-3’ |
|  | reverse 5’-TCCGCTCTCTGTCAAAGTGTG-3’ |
| Mouse SOD1 | forward 5’-AACCAGTTGTGTTGTCAGGAC-3’ |
|  | reverse 5’-CCACCATGTTTCTTAGAGTGAGG-3’ |
| Mouse SOD2 | forward 5’-CAGACCTGCCTTACGACTATGG-3’ |
|  | reverse 5’-CTCGGTGGCGTTGAGATTGTT-3’ |
| Mouse SOD3 | forward 5’-CCTTCTTGTTCTACGGCTTGC-3’ |
|  | reverse 5’-TCGCCTATCTTCTCAACCAGG-3’ |
| Mouse CD68 | forward 5’-TGTCTGATCTTGCTAGGACCG-3’ |
|  | reverse 5’-GAGAGTAACGGCCTTTTTGTGA-3’ |
| Mouse CD86 | forward 5’-TCAATGGGACTGCATATCTGCC-3’ |
|  | reverse 5’-GCCAAAATACTACCAGCTCACT-3’ |
| Mouse CCR7 | forward 5’-ATGACGTCACCTACAGCCTG-3’ |
|  | reverse 5’-CAGCCCAAGTCCTTGAAGAG-3’ |
| Mouse IL-4 | forward 5’-GGTCTCAACCCCCAGCTAGT-3’ |
|  | reverse 5’-GCCGATGATCTCTCTCAAGTGAT-3’ |
| Mouse IL-10 | forward 5’-GCTCTTACTGACTGGCATGAG-3’ |
|  | reverse 5’-CGCAGCTCTAGGAGCATGTG-3’ |
| Mouse CD163 | forward 5’-TGGGTGGGGAAAGCATAACT-3’ |
|  | reverse 5’-AAGTTGTCGTCACACACCGT-3’ |
| Mouse CD206 | forward 5’-AGACGAAATCCCTGCTACTG-3’ |
|  | reverse 5’-CACCCATTCGAAGGCATTC-3’ |
| Mouse TGF-β | forward 5’-CAGTACAGCAAGGTCCTTGC-3’ |
|  | reverse 5’-ACGTAGTAGACGATGGGCAG-3’ |
| Mouse Arg1 | forward 5’-CTCCAAGCCAAAGTCCTTAGAG-3’ |
|  | reverse 5’-GGAGCTGTCATTAGGGACATCA-3’ |
| Mouse Fizz1 | forward 5’-CCTGCTGGGATGACTGCTACT-3’ |
|  | reverse 5’-AGATCCACAGGCAAAGCCAC-3’ |
| Mouse MMP3 | forward 5’-ACATGGAGACTTTGTCCCTTTTG-3’ |
|  | reverse 5’-TTGGCTGAGTGGTAGAGTCCC-3’ |
| Mouse MMP13 | forward 5’-CTTCTTCTTGTTGAGCTGGACTC-3’ |
|  | reverse 5’-CTGTGGAGGTCACTGTAGACT-3’ |
| Mouse IL-1β | forward 5’- CCCAACTGGTACATCAGCACCTC-3’ |
|  | reverse 5’- GACACGGATTCCATGGTGAAGTC-3’ |
| Mouse IL-6 | forward 5’- ATAGTCCTTCCTACCCCAATTTCC-3’ |
|  | reverse 5’- GATGAATTGGATGGTCTTGGTCC-3’ |
| Mouse TNF-α | forward 5’- CTGAACTTCGGGGTGATCGG-3’ |
|  | reverse 5’- GGCTTGTCACTCGAATTTTGAGA-3’ |

Additional Figures


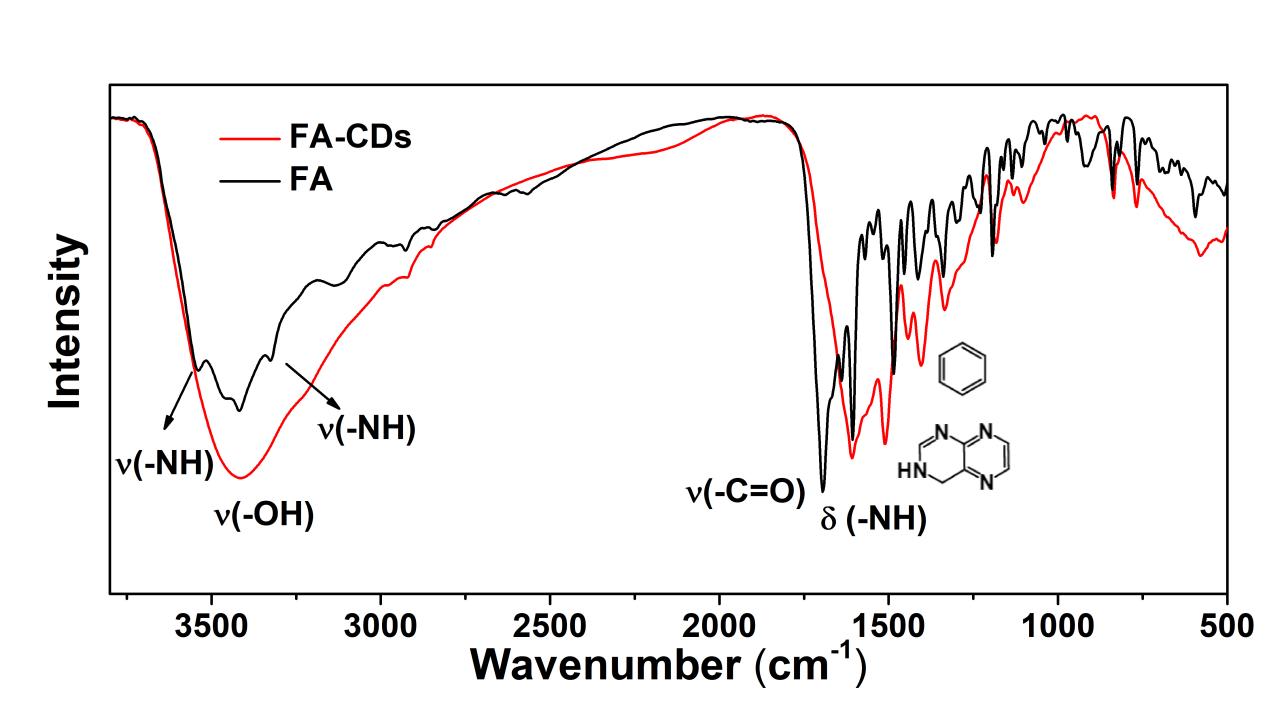


Figure S1. FTIR spectra of folic acid (FA) and folic acid derived carbon dots (FA-CDs) prepared

in this research.


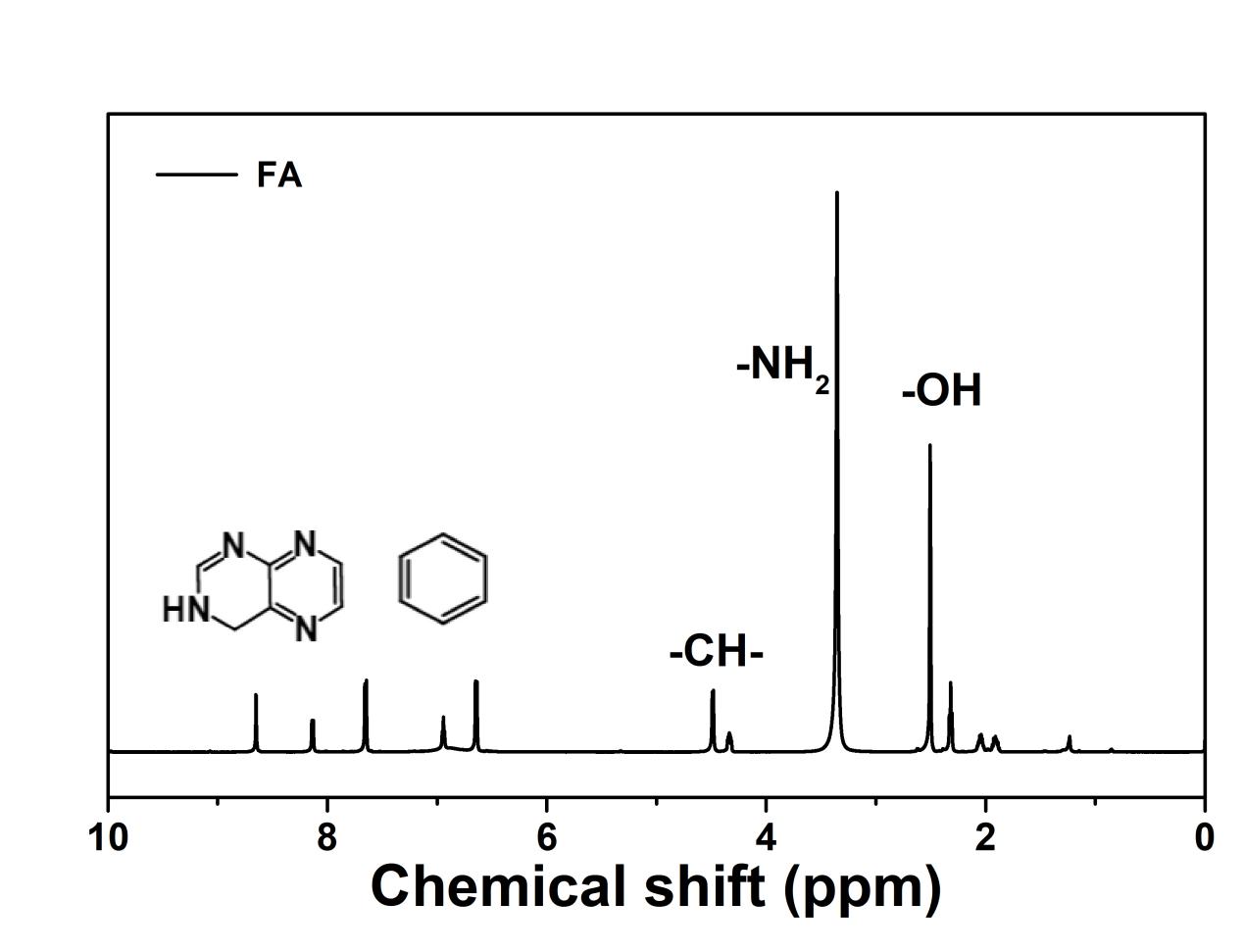


Figure S2. H^1^ NMR spectra of FA.


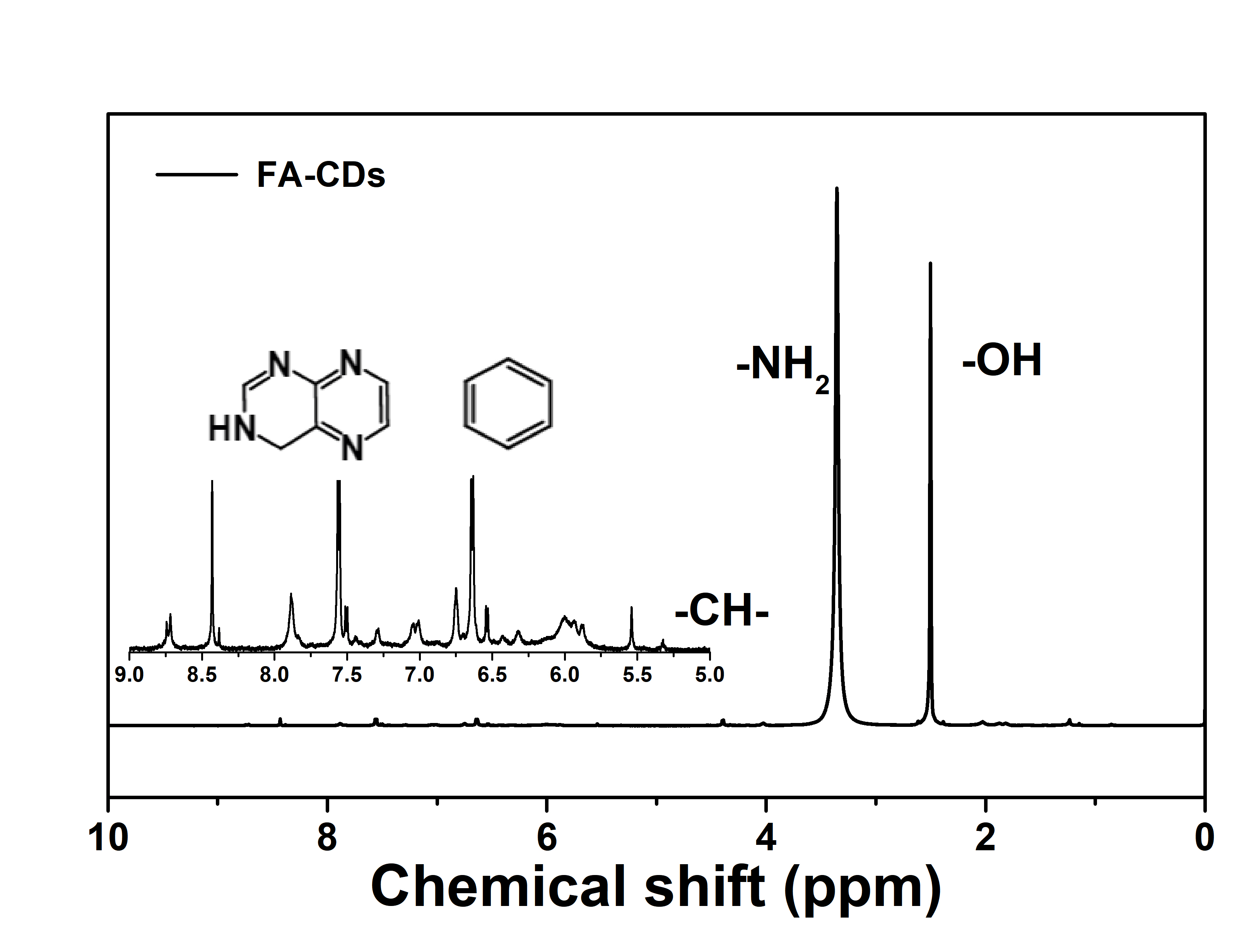


Figure S3. H^1^ NMR spectra of FA-CDs.


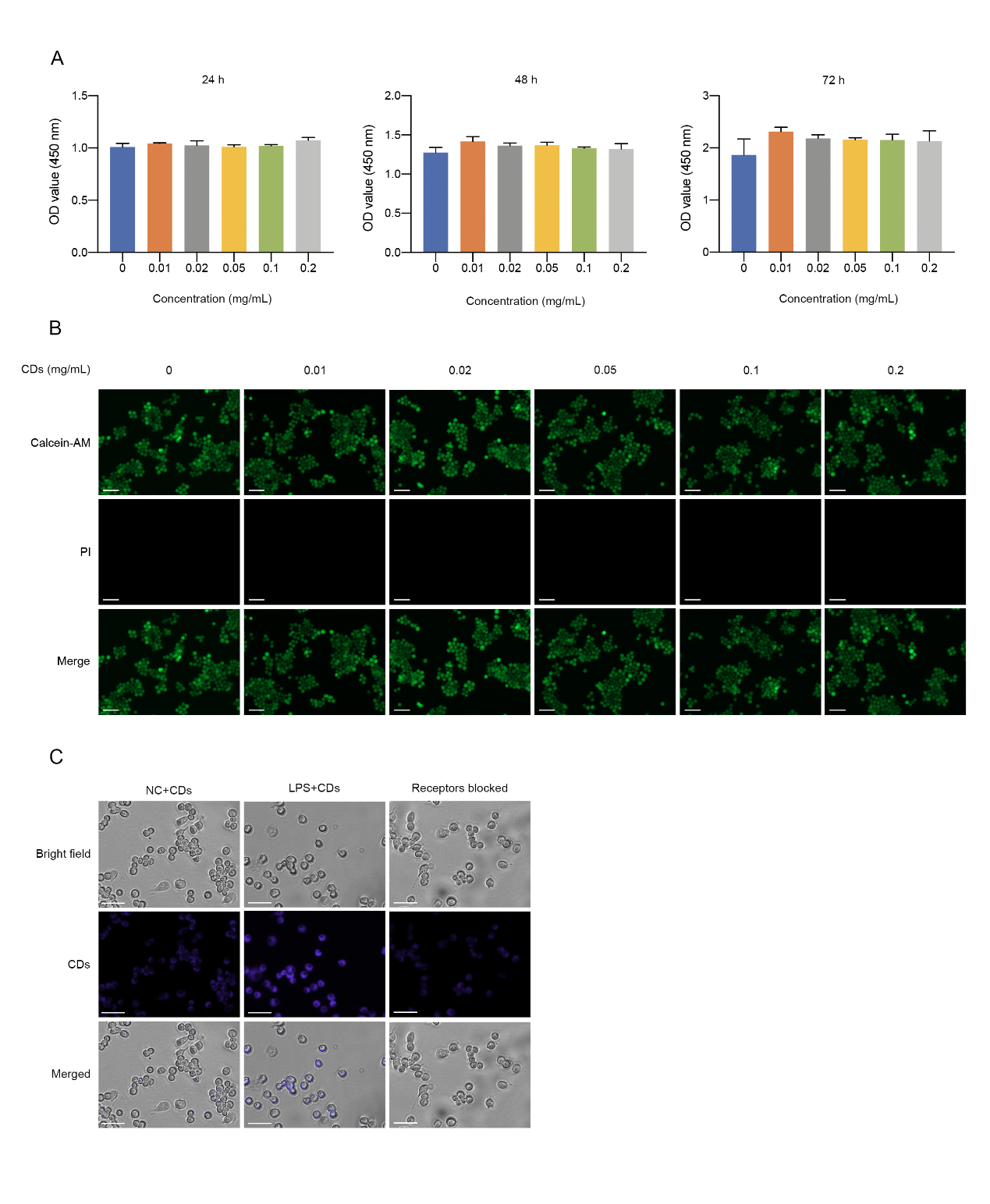
Figure S4. Biocompatibility and cellular uptake CDs in RAW264.7 cells.

(A) CCK8 assay of RAW264.7 cells after incubation with different concentration of CDs for 24 h, 48 h, and 72 h. (B) Live & dead staining of RAW264.7 cells incubated with different concentration of CDs for 24 h. Scar bar: 40 μm (C) Cellular uptake of CDs in RAW264.7 cells with or without LPS stimulation, or LPS-induced RAW264.7 cells with FRs pre-blocked.

Scar bar: 50 μm
